# Hepatoprotective effects of empagliflozin in metabolic dysfunction-associated steatotic liver disease: a systematic review and meta-analysis of preclinical studies

**DOI:** 10.3389/fphar.2026.1822157

**Published:** 2026-07-24

**Authors:** Lashin S. Ali, Mohamed O. Mostafa, Dana Alsharayiah, Mohamed A. Elkholy, Khaled A. Ahmed, Ahmed A. El-Mansy, Abd El Rahman M. Sharfeldeen, Gehan El Wakeel, Abdelnaser A. Badawy, Ahmed H. Sulaiman, Elryah I. Ali, Mariam Shabram Alenazi, Mohammed M. Alruwaili, Eman Hamza, Mamdouh Eldesoqui

**Affiliations:** 1 Department of Basic Dental Science, Faculty of Dentistry, Al-Ahliyya Amman University, Amman, Jordan; 2 Department of Medical Physiology, Mansoura Faculty of Medicine, Mansoura University, Egypt; 3 Department of Oral Pathology, Faculty of Dentistry, Beni-Suef university, Beni-Suef, Egypt; 4 Department of Basic and Clinical Medical Sciences, Faculty of Dentistry, Zarqa University, Zarqa, Jordan; 5 Department of Medical Histology and Cell Biology, Faculty of Medicine, Mansoura University, Mansoura, Egypt; 6 Department of Oral and Maxillofacial Pathology, College of Dentistry, City University Ajman, Ajman, UAE; 7 Department of Oral and Maxillofacial Pathology, Faculty of Dentistry, Assiut University, Assiut, United Egypt; 8 Department of Basic Dental Sciences, Faculty of Dentistry, The Hashemite University, Zarqa, Jordan; 9 Department of Biochemistry, Faculty of Medicine, Northern Border University, Arar, Saudi Arabia; 10 Department of Medicine, Faculty of Medicine, Northern Border University, Arar, Saudi Arabia; 11 Department of Medical Laboratory Technology, College of Applied Medical Sciences, Northern Border University, Arar, Saudi Arabia; 12 Department of Family & Community Medicine, Faculty of Medicine, Northern Border University, Arar, Saudi Arabia; 13 Center for Health Research, Northern Border University, Arar, Saudi Arabia; 14 Department of Biochemistry, College of Medicine, Imam Mohammad Ibn Saud Islamic University (IMSIU), Arar, Saudi Arabia; 15 Department of Basic Medical Sciences, College of Medicine, AlMaarefa University, Dariyah, Saudi Arabia; 16 Research Center, Deanship of Scientific Research and Post-Graduate Studies, AlMaarefa University, Dariyah, Saudi Arabia

**Keywords:** Empaglifiozin, Fibrosis, Hepatoproctective effects, MASH, MASLD, Sodium-glucose cotransoporter-2 inhibitors

## Abstract

**Background:**

The worldwide burden of metabolic dysfunction-associated steatotic liver disease (MASLD) is rising sharply, powered by high prevalence of obesity and its associated metabolic insults. Though urgent, definitive treatment is not yet available. Empagliflozin, a sodium glucose co-transporter inhibitor has been suggested in both human and animal studies to have diverse beneficial effects in MASLD by anti-inflammation and anti-oxidative stress effects, but many of the underlying mechanisms remain unexplained. The main aim of this systematic review and meta-analysis was to evaluate the effects of empagliflozin’s in preclinical animal studies trying to view new horizons for future treatment of MASLD.

**Methods:**

we searched the related animal preclinical studies in databases including Scopus, Web of Science, Cochrane library, Wily online library and PubMed. The research team screened the literature and extracted data; any discrepancies were resolved by the research team leader through discussion. The quality of research methods was assessed by SYRCLE (systematic review center for laboratory animal experimentation) risk of bias tool. The meta-analysis was guided by the Cochrane Handbook; statistical analyses were conducted via RevMan 5.4 offline version and Comprehensive Met analysis V3 software.

**Results:**

Our meta-analysis included 17 studies, involving 584 animals. In comparison with the model group, empagliflozin significantly reduced steatosis markers including hepatic burden of lipids, including triglycerides and cholesterol, NAFLD Activity Score (NAS) and total serum cholesterol (TC). It also significantly reduced hepatic fibrosis markers including fibrosis grade, transforming growth factor-β (TGF-β) and collagen type I alpha 1 chain) COL1A1). Beyond its anti-steatofibrotic effects, empagliflozin significantly downregulated hepatic expression of inflammatory and oxidative markers including tumor necrosis factor-alpha (TNF-α), interleukin-6 (IL-6) and malondialdehyde (MDA). Moreover, empagliflozin significantly improved glycemic indices including Homeostatic Model Assessment for Insulin Resistance (HOMA-IR), serum insulin, and fasting blood sugar (FBS).

**Conclusion:**

Empagliflozin significantly protected the liver against steatosis and fibrosis by mitigating hepatic inflammation, oxidative stress and insulin resistance.

## Introduction

Metabolic dysfunction–associated Steatotic liver disease (MASLD) is the new name for non- alcoholic fatty liver disease (NAFLD) since 2023, represents a progressive disease spectrum, that extends from simple hepatic steatosis to steatohepatitis, fibrosis, cirrhosis, and hepatocellular carcinoma (HCC) in its late stages (Rinella et al., 2023). Given its strong association with cardiometabolic risk factors, MASLD has emerged as a major global public health challenge, carrying substantial worldwide clinical and economic burdens (Younossi et al., 2024).

The global impact of MASLD has reached critical proportions, with recent estimates from 2025 indicating a worldwide prevalence of approximately 38% among adults (Danpanichkul et al., 2025). Beyond its clinical impact as the leading cause of chronic liver disease, MASLD imposes a staggering economic strain**.** Furthermore, MASLD is now recognized as a primary indication for liver transplantation, especially among women and patients with concurrent type 2 diabetes, highlighting a pressing need for prioritized public health interventions (Miao et al., 2024). Now, it is widely accepted that MASLD is a multisystemic metabolic disorder with profound extrahepatic consequences, especially on cardiovascular disease (Tarantino et al., 2014; Cliff et al., 2026) which remains a leading cause of morbidity and mortality among patients with MASLD.

While recent therapeutic advances, including the approval of resmetirom by the FDA in 2024, and the established efficacy of semaglutide (Wegovy) (Dichtel, 2021; Newsome et al., 2021), represent a marked progress in the management of MASLD, the underlying molecular pathophysiologic mechanisms of hepatic protection remain incompletely understood. Preclinical investigations continue to play an essential role in revealing these pathways.

Empagliflozin, a potent and selective sodium-glucose cotransporter-2 (SGLT2) inhibitor, has been introduced as a pivotal pharmacological intervention for patients presenting with MASLD complicated by type 2 diabetes**.** This agent facilitates a dual-targeting therapeutic approach by concurrently modulating renal glucose handling and hepatic metabolic pathways. By decreasing the proximal renal tubular glucose reabsorption, empagliflozin promotes glucose loss in urine, which subsequently drives significant weight reduction and optimizes glycemic homeostasis (Shojaei et al., 2025). Beyond systemic glucose lowering, its pleiotropic profile encompasses enhanced peripheral insulin sensitivity (Alqudah et al., 2024), suppression of hepatic gluconeogenesis, renal protection (Ramadan et al., 2025) and the upregulation of fatty acid oxidation, collectively mitigating hepatic steatosis and metabolic dysregulation (Comella et al., 2025).

Recent clinical evidence underscores that the hepatoprotective efficacy of empagliflozin extends far beyond simple glucose reduction. The medication attenuates hepatic lipid accumulation through a multifaceted mechanism involving the downregulation of *de novo* lipogenesis, stimulation of lipolysis, and restoration of mitochondrial bioenergetics (Heo et al., 2025). Moreover, the anti-inflammatory and anti-fibrotic effects of empagliflozin are crucial in reducing the progression of Metabolic Dysfunction-Associated Steatohepatitis (MASH) and subsequent hepatic fibrosis and cirrhosis (Fu et al., 2025).

Despite the abundance of meta-analyses evaluating the clinical impact of empagliflozin, there remains a marked scarcity of evidence syntheses focusing on preclinical research of SGLT-2i. To date, there are no systematic reviews with meta-analyses quantifying empagliflozin’s effects in preclinical models of MASLD. We aimed to address this gap by conducting this comprehensive systematic review and meta-analysis of experimental studies which provides a robust preclinical framework to inform future experimental designs and guide clinical translation.

## Literature search and review strategy

Our systematic review and meta-analysis followed Cochrane Handbook guidelines for systematic reviews. The included preclinical animal studies published between 2016 and October 2025, were searched in Scopus, Wily Online Library, PubMed, Web of Science and Cochrane Library

The medical subject terms (MeSH) and free terms used for database searches are: (““Empagliflozin”” or ““Jardiance”” or ““Sodium-Glucose Transporter 2 inhibitor”” or ““SGLT-2 inhibitor”” OR “BI 10773”) AND (““Non-alcoholic Fatty Liver Disease”” or ““Nonalcoholic Fatty Livers”” or ““NAFLD”” or ““Nonalcoholic Steatohepatitis”” or ““NASH”” or ““metabolic dysfunction-associated steatotic liver disease”” or ““metabolic dysfunction associated steatohepatitis”” or ““MASLD”” or ““MASH””). The search strategy was modified to adapt each data base.

### Inclusion criteria

Selection of relevant studies from preclinical literature followed these inclusion criteria 1) randomized controlled trials, 2) animal models of MASLD or MASH with or without fibrosis, 3) animal models limited to male mice and rats, 4) empagliflozin doses ≤ 30 mg/kg/day, 5) empagliflozin was introduced through gavage or with diet, 6) intervention duration of minimum 4 weeks, 7) primary outcome results must include one or more of the followings: hepatic triglycerides, hepatic cholesterol, oil red area, hepatocyte ballooning, NAS, fibrosis grade TGF-β, (COL1A1), α-SMA,ALT (alanine aminotransferase) and AST (aspartate aminotransferase), 8) secondary outcome results must include one or more of the following: TNF-α,IL-6, lobular inflammation, MDA, nuclear factor erythroid 2-related factor 1)Nrf1), catalase, fasting blood sugar (FBS), HOMA-IR, body weight and liver weight

### Exclusion criteria

1) Clinical trials, 2) *In vitro* cell and tissue studies, 3) Other animal species, 4) Diabetic rat models, 5) Cirrhosis models, 6) non-English publications

### Study selection and data extraction

After retrieval of records from electronic databases, duplicate citations were removed using Zotero reference management software. Three researchers performed the initial screening independently based on titles and abstracts according to inclusion and exclusion criteria. This was followed by full-text assessment to determine final inclusion. Study selection was conducted independently by all researchers, the final decision in discrepancies was taken by the research team leader after discussion.

Results were expressed as continuous variables; therefore, the mean and corresponding standard deviation for both intervention and control groups were extracted. When data were reported as mean ± standard error of the mean (SEM), values were converted to mean ± SD using appropriate statistical formulas based on the sample size of each group.

For studies in which outcomes were presented solely in graphical form, corresponding authors were contacted by e-mail to obtain the numerical data. When such data could not be obtained, values were independently extracted from graphs using WebPlotDigitizer software by two independent researchers, with discrepancies resolved by the research team leader.

### Statistical analysis

Our systematic review and meta-analysis followed PRISMA guidelines. Statistical analyses were performed using Comprehensive Meta-Analysis (CMA) version 3 and Review RevMan 5.4 software. As all outcomes were continuous data, pooled effect sizes were calculated as standardized mean differences (SMDs) with corresponding 95% confidence intervals (CIs). Heterogeneity between studies was assessed using the I^2^ statistic, and owing to substantial heterogeneity, a random-effects model was applied.

To investigate the possible causes of heterogeneity, predefined subgroups were designed for primary outcomes based on dosage (≤10 mg/kg/day vs. >10 mg/kg/day), animal species (mice vs. rats), and intervention duration (≤5 weeks vs. >5 weeks).

Sensitivity analyses for robustness of outcomes were performed using a leave-one-out approach. For outcomes including ten or more studies, publication bias was assessed through visual inspection of funnel plots and quantitatively evaluated using Egger’s regression test. When publication bias was identified, as indicated by funnel plot asymmetry and an Egger’s test, the trim-and-fill method was applied to investigate the pooled effect estimates.

## Results

### A-Research screening

The database research identified a total of 1,127 records, including 108 from the Cochrane Library, 135 from Wiley Online Library, 123 from PubMed, 595 from Scopus, and 166 from Web of Science. Following removal of duplicate records, 816 studies remained for screening.

**FIGURE 1:**
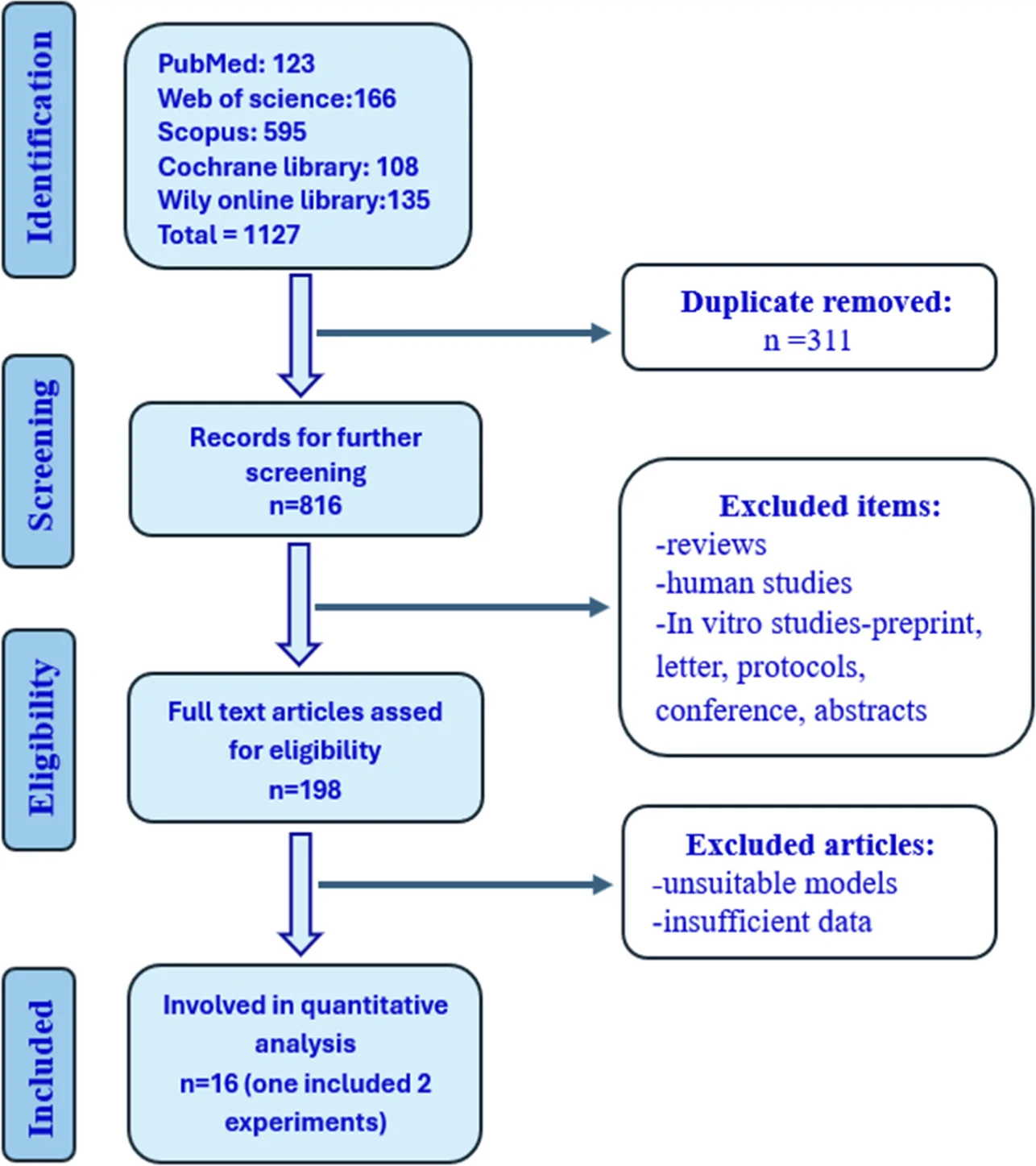
PRISMA flow chart.

Titles and abstracts were initially screened, followed by full-text assessment in accordance with the predefined eligibility criteria. Ultimately, 16 articles met the inclusion criteria and were joined in our systematic review and meta-analysis. One included article (Kim et al., 2023) included two independent experimental datasets. Selection process of the included articles is summarized in the PRISMA flow graph (Figure 1).

### B-Study characteristics

The spectrum of the 17 studies included 584 animals, and 251 animals were involved in the control and intervention groups. The animal species included mice and rats, thirteen studies of all which used C57BL/6 mice (Xu et al., 2017; Petito-da-Silva et al., 2019; Nasiri-Ansari et al., 2021; Perakakis et al., 2021; Heo et al., 2022; Kurtz et al., 2022; Lee et al., 2022; Ma et al., 2022; Kim et al., 2023; Niu et al., 2023; Radlinger et al., 2023; Fu et al., 2025; Heo et al., 2025), three studies used rats (Abdalla et al., 2023; Elseweidy et al., 2024; Zambrano-Vásquez et al., 2025). The studies featured six animal models of high-energy diet: high fat diet (HFD), choline-deficient, L-amino acid defined, high-fat diet (CDAHFD), Western-type diet (WD), GAN diet, AMLN diet and high milk-fat diet. The empagliflozin dosages in these studies ranged from 8 to 30 mg/kg/day through gavage or with diet, but most studies utilized a dose of 10mg/kg/ day. Duration of modeling ranged from 4 to 16 weeks, but most studies lasted for 5 weeks (Table 1).

**TABLE 1:** Study characteristics.

Study	Year	Species, (number, treatment/control), age	Models (method, duration)	Empagliflozin (dose, route, duration)	Outcomes
Lee et al. (2022)	2022	C57BL/6J mice, n=8/8, 7 weeks	CDAHFD, 5 weeks	10mg/kg/day, by gavage,5 weeks	NAS, TGF-*β*, COL1α1, αSMA, TNF-α, IL-6, F40/80, MDA, catalase
Petito-da-Silva et al. (2019)	2019	C57BL/6 mice, n=10/10, 12 weeks	HFD, 10 weeks	10 mg/kg/day, mixed with diet,5 weeks	Liver TG, HOMA-IR, Insulin, ALT, TG, TC
Nasiri-Ansari et al. (2021)	2021	C57BL/6J mice, n= 8/8, 5 weeks	HFD, 5 weeks	10 mg/kg/day, by gavage,5 weeks	NAS, Glucose, ALT, AST, TG, TC
Radlinger et al. (2023)	2023	C57BL/6 mice, 7 weeks	WD, 10 weeks	30 mg/kg/day, with diet,10 weeks	Liver TG, Insulin, Glucose
Elseweidy et al. (2023)	2023	Wistar albino rats, n= 6/6	HFD, 10 weeks	30 mg/kg/day orally, 8 weeks	HOMA-IR, Insulin, Glucose, ALT, AST, TG, TC
Fu et al. (2025)	2025	C57BL/6J mice, n= 6/6, 5 weeks	GAN diet 5 weeks	8 mg/kg/day, by gavage,5 weeks	ALT, AST, TG, TC
Xu et al. (2017)	2017	C57BL/6J mice, n=8/8, 8 weeks	HFD, 16 weeks	10 mg/kg/day, mixed with diet, 16 weeks	Insulin, Glucose, ALT, AST, TG, TC
Perakakis et al. (2021)	2021	C57BL/6JRj mice, n= 13/13	AMLN diet 12 weeks	10 mg/kg/day, mixed with diet, 12 weeks	HOMA-IR, Insulin, Glucose, ALT, AST, TG, TC
Heo et al. (2025)	2025	C57BL/6J mice, n=8/8, 8 weeks	HFD, 18 weeks	10 mg/kg day, orally, 18 weeks	Liver TG, Glucose, ALT, AST
Zambrano-Vásquez et al. (2025)	2025	Wistar rats, n= 6/6,4 weeks	Western-type diet 30 days	12.5 mg/kg/day, by gavage, 30 days	MDA, Glucose, ALT, AST, TG, TC
Abdalla et al. (2023)	2023	Albino rats, n= 8/8, 5 weeks	CCl4, 8 weeks	10 mg/kg/day, by gavage,8 weeks	TGF-*β*, αSMA, TNF-α, IL-6, MDA, ALT, AST
Kim et al. (2023).a	2023	C57BL/6 N mice, n= 5/5, 6 weeks	CDAHFD, 8 weeks	10 mg/kg/day, by gavage,8 weeks	Liver TG, Liver C, NAS, Glucose, ALT, AST, TG, TC
Kim et al. (2023).b	2023	C57BL/6 N mice, n= 5/5, 6 weeks	CDAHFD, 30 weeks	10 mg/kg/day, by gavage,12 weeks	Liver TG, Liver C, NAS, Glucose, ALT, AST, TG, TC
Heo et al. (2022)	2022	C57BL/6J mice, n=8/8, 5 weeks	CDAHFD, 5 weeks	10 mg/kg/day, by gavage, 5 weeks	TGF-*β*, αSMA
Niu et al. (2023)	2023	C57BL/6J mice, n= 9/9, 6 weeks	HFD, 12 weeks	10 mg/kg/day, by gavage, 6 weeks	TNF-α, IL-6, MDA, Glucose, ALT, AST, TG, TC
Ma et al. (2022)	2023	C57BL/6J mice, n=6/6, 6 weeks	HFD, 12 weeks	10 mg/kg/day, by gavage, 8 weeks	Liver TG, TNF-α, MDA, Glucose, ALT, AST, TG
Kurtz et al. (2022)	2022	C57BL6J mice, n=6/6, 12-14 weeks	HMFD, 12 weeks	10 mg/kg/day, mixed with diet,12 weeks	Liver TG, Liver C, Glucose

### C-Study quality and risk of bias

**FIGURE 2:**
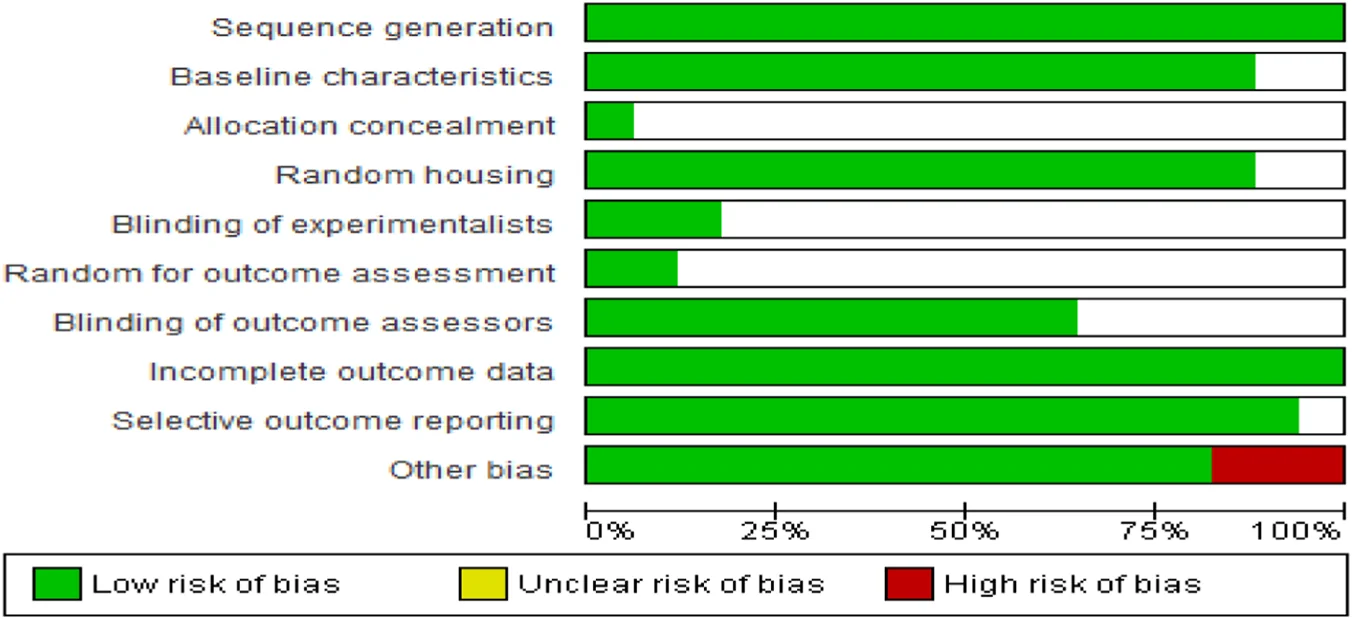
Graphic representation of risk of bias.

The quality of the studies involved in our meta-analysis was assessed using the SYRCLE risk of bias tool for animal studies (Table 2; Figure 2). The SYRCLE tool includes 10 items within the 6 main domains (Hooijmans et al., 2014): 1) selection bias domain which includes 3 items, sequence generation (A), baseline characteristics (B) and allocation concealment (C), 2) Performance bias domain which includes 2 items, random housing (D) and blinding of experimentalists (E), 3) Detection bias domain which includes 2 items, random for outcome assessment (F) and blinding of outcome assessors (G), 4) Attribution bias domain, incomplete outcome data (H), 5) Reporting bias domain, selective outcome reporting (I), 6) Other bias domain (J).

**TABLE 2:** SYRCLE risk of bias tool for the included studies.

Study	A	B	C	D	E	F	G	H	I	J
Lee et al. (2022)	**+**	**+**	**?**	**+**	**+**	**?**	**+**	**+**	**+**	**+**
Petito-da-Silva et al. (2019)	**+**	**+**	**?**	**+**	**?**	**?**	**+**	**+**	**+**	**+**
Nasiri-Ansari et al. (2021)	**+**	**+**	**?**	**+**	**?**	**?**	**+**	**+**	**+**	**+**
Radlinger et al. (2023)	**+**	**+**	**?**	**+**	**?**	**?**	**+**	**+**	**+**	**+**
Elseweidy et al. (2023)	**+**	**+**	**?**	**?**	**?**	**?**	**+**	**+**	**+**	**+**
Fu et al. (2025)	**+**	**+**	**?**	**+**	**?**	**?**	**+**	**+**	**+**	**+**
Xu et al. (2017)	**+**	**+**	**?**	**+**	**?**	**?**	**?**	**+**	**+**	**-**
Perakakis et al. (2021)	**+**	**+**	**?**	**?**	**?**	**?**	**+**	**+**	**+**	**+**
Heo et al. (2025)	**+**	**+**	**?**	**+**	**+**	**+**	**+**	**+**	**+**	**+**
Zambrano-Vásquez et al. (2025)	**+**	**+**	**+**	**+**	**+**	**+**	**+**	**+**	**+**	**+**
Abdalla et al. (2023)	**+**	**?**	**?**	**+**	**?**	**?**	**?**	**+**	**?**	**+**
Kim et al. (2023).a	**+**	**+**	**?**	**+**	**?**	**?**	**?**	**+**	**+**	**-**
Kim et al. (2023).b	**+**	**+**	**?**	**+**	**?**	**?**	**?**	**+**	**+**	**-**
Heo et al. (2022)	**+**	**?**	**?**	**+**	**?**	**?**	**?**	**+**	**+**	**+**
Niu et al. (2023)	**+**	**+**	**?**	**+**	**?**	**?**	**+**	**+**	**+**	**+**
Ma et al. (2022)	**+**	**+**	**?**	**+**	**?**	**?**	**?**	**+**	**+**	**+**
Kurtz et al. (2022)	**+**	**+**	**?**	**+**	**?**	**?**	**+**	**+**	**+**	**+**

Sequence generation (A), baseline characteristics (B), allocation concealment (C), random housing (D), blinding of experimentalists (E), random for outcome assessment (F), blinding of outcome assessors (G), incomplete outcome data (H), selective outcome reporting (I), Other bias domain (J).

Risk of bias was evaluated by two independent researchers with discrepancies resolved by the research team leader. Each item was evaluated as high risk, low risk or unclear (Table 2). In summary, studies were judged as low risk (+), unclear risk (?) or high risk (-). The overall evaluation of the ten items of SYRCLE tool was 64.1% for low risk of bias, 34.1% for unclear risk of bias and 1.8% for high risk of bias. The highest values for low risk of bias were seen in sequence generation (100% of studies), incomplete outcome data (100% of studies), selective outcome reporting (94.2% of studies), baseline characteristics (88.3% of studies), random housing (88.3% % of studies) and blinding of outcome assessors (64.7% of studies).

In contrast, low risk of bias was lower in blinding of experimentalists (17.6% of studies). Random outcome assessment (11.7% of studies) and allocation concealment (5.8% of studies), with the remaining studies evaluated as unclear risk. Regarding other biases, most studies (82.4%) were evaluated as low risk, while a small proportion (17.6%) were evaluated as high risk.

## Primary outcome measure

### Effect of empagliflozin on hepatic tissue lipids and serum lipids

Lipid accumulation above physiological limits is a hallmark of MASLD and is considered the most important factor determining the speed of progression to MASH (Carli et al., 2024).

**FIGURE 3:**
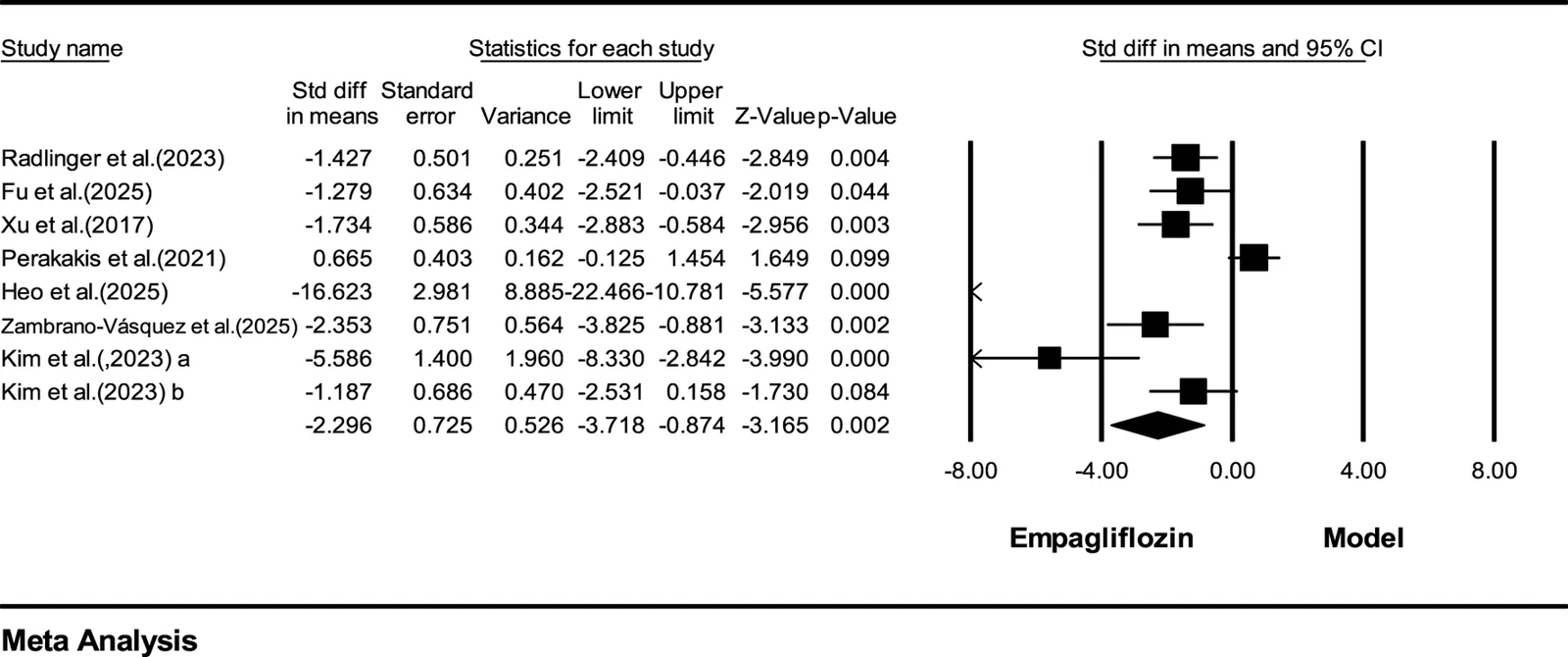
Hepatic TG (*mg/gm*).

**FIGURE 4:**
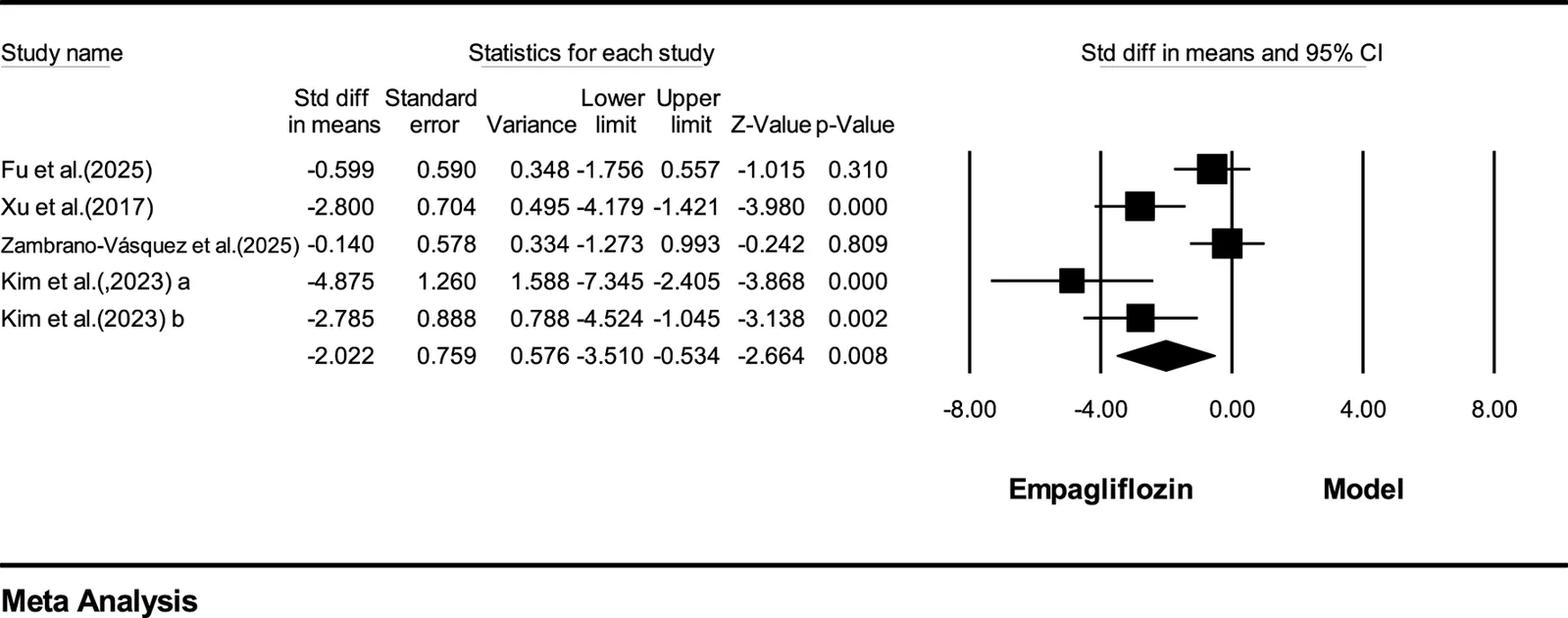
Hepatic cholesterol (mg/gm).

The main markers of intra-hepatic lipid accumulation, hepatic tissue triglycerides and hepatic tissue cholesterol are evaluated in our meta-analysis. Hepatic tissue triglycerides are assessed in eight studies and hepatic tissue cholesterol is assessed in five studies. Both are significantly reduced in the empagliflozin groups compared to the model groups, for hepatic tissue triglycerides [SMD = −2.296 (95% CI: -3.718 to −0.874); heterogeneity: I^2^ = 88.52%, P < 0.05] (Figure 3) and for hepatic tissue cholesterol [SMD = −2.022 (−95% CI: 3.510 to −0.534); heterogeneity: I^2^ = 80.52%, P < 0.05] (Figure 4).

**FIGURE 5:**
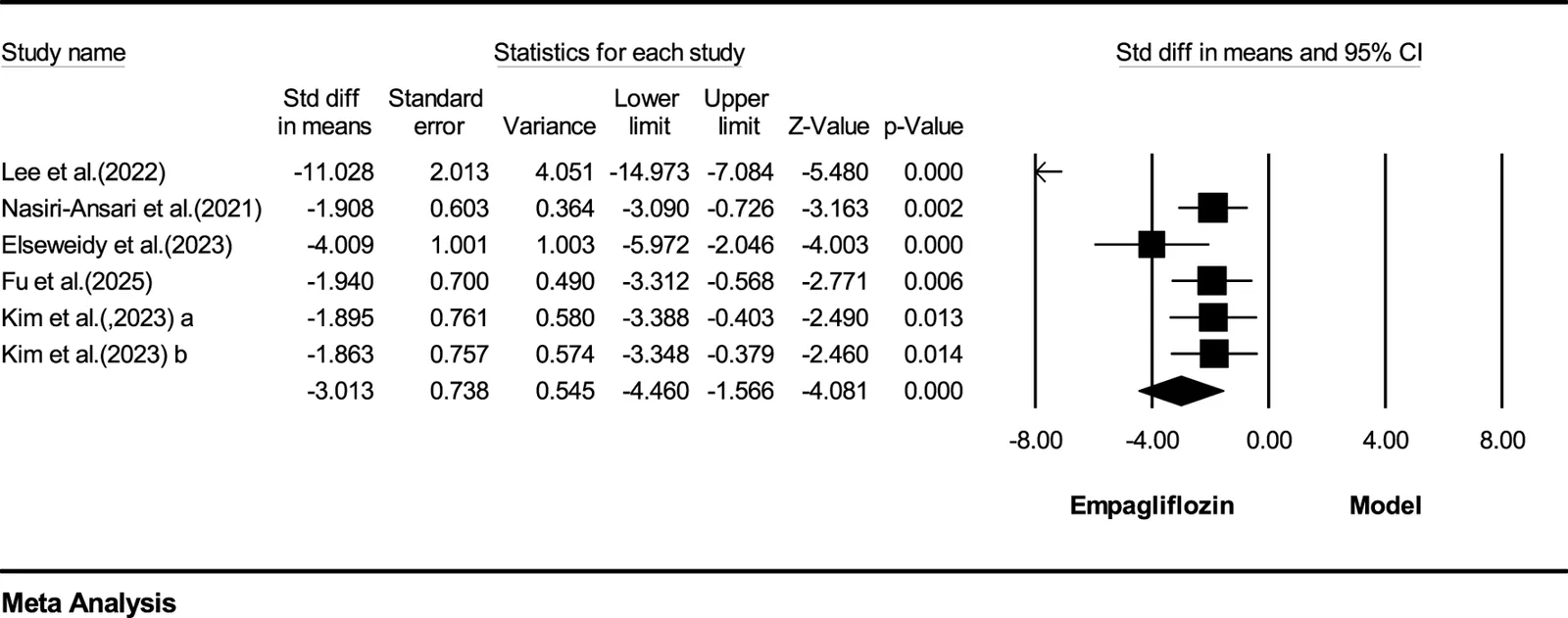
NAS.

**FIGURE 6:**
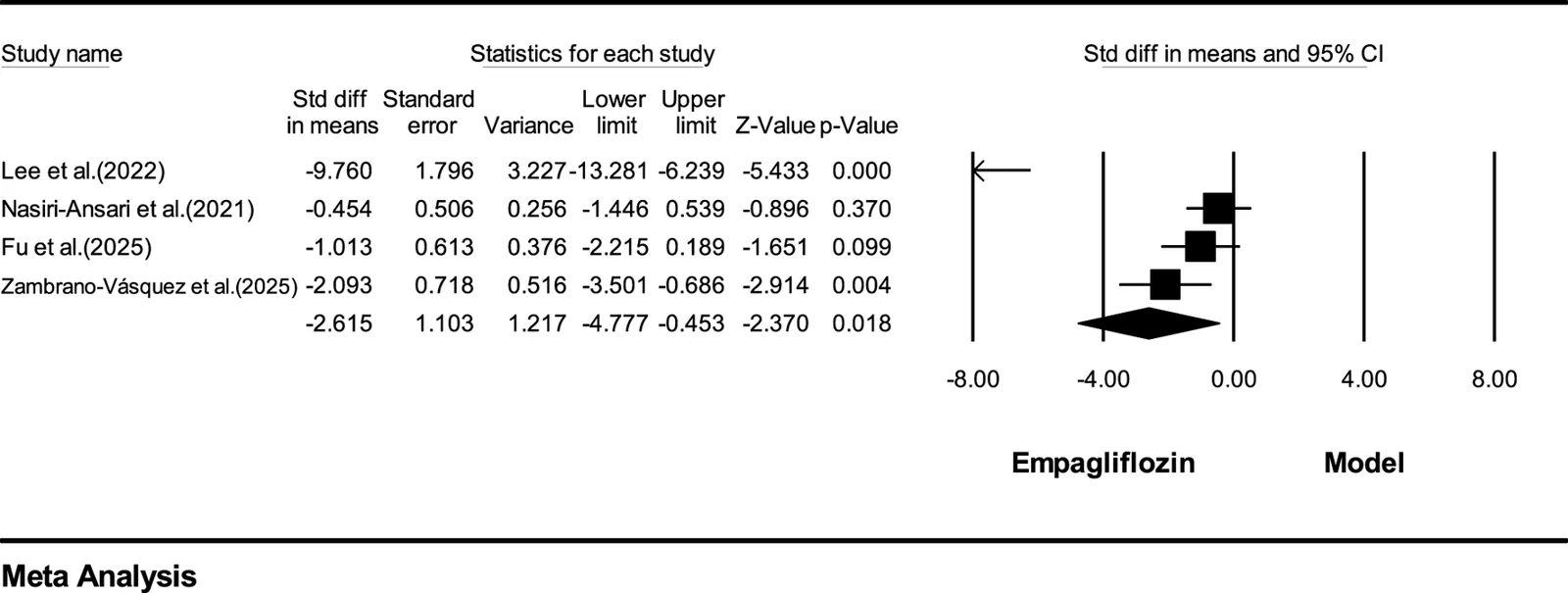
Hepatocyte ballooning.

**FIGURE 7:**
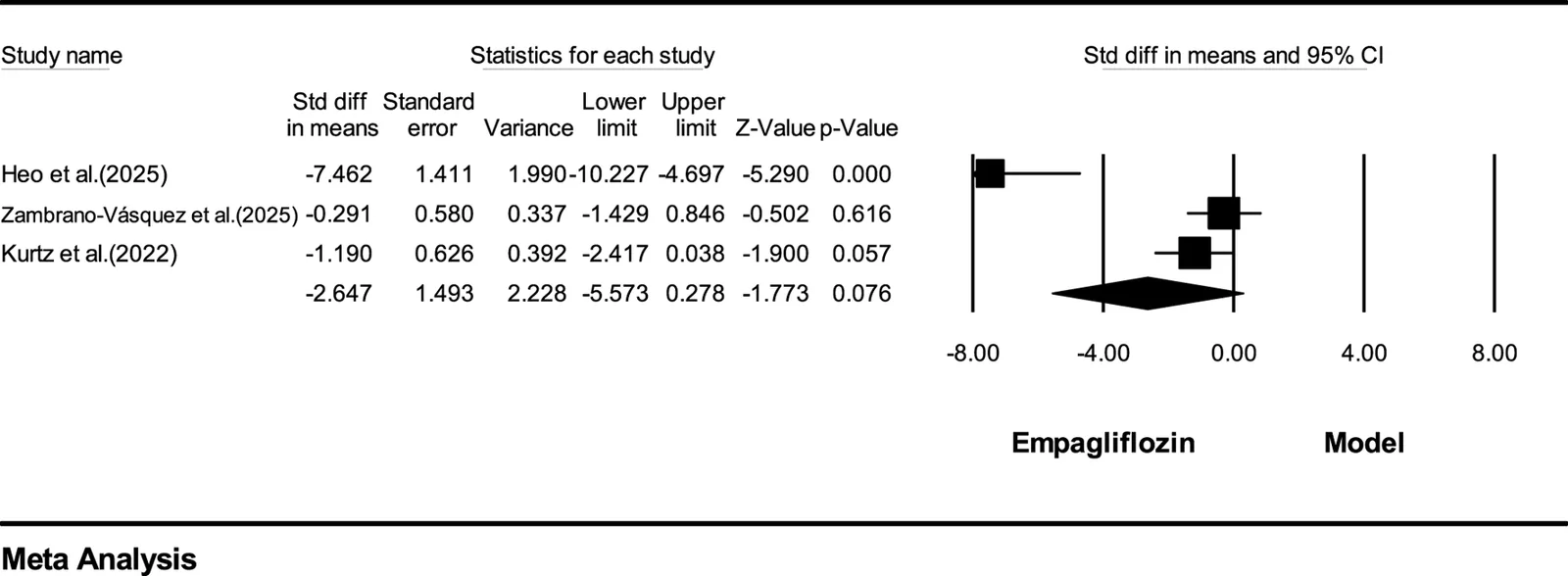
Oil red area.

Hepatic histopathology is evaluated by NAS, hepatocyte ballooning and oil red area, six studies assessed NAS, four studies assessed hepatocyte ballooning, and three studies assessed oil red area. NAS and Hepatocyte ballooning are significantly reduced in the empagliflozin groups when compared to the model groups, for NAS [SMD = −3.013 (95% CI: -4.460 to −1.566); heterogeneity: I^2^ = 78.24%, P < 0.05] (Figure 5) and for hepatocyte ballooning [SMD = −2.615 (95% CI: -4.777 to −0.453); heterogeneity: I^2^ = 88.64%, P < 0.05] (Figure 6). However oil red area was insignificantly reduced in the empagliflozin groups when compared to the model groups [SMD = −2.64 (95% CI: -5.57 to −0.27]; heterogeneity: I^2^ = 90.95%, P > 0.05] (Figure 7).

**FIGURE 8:**
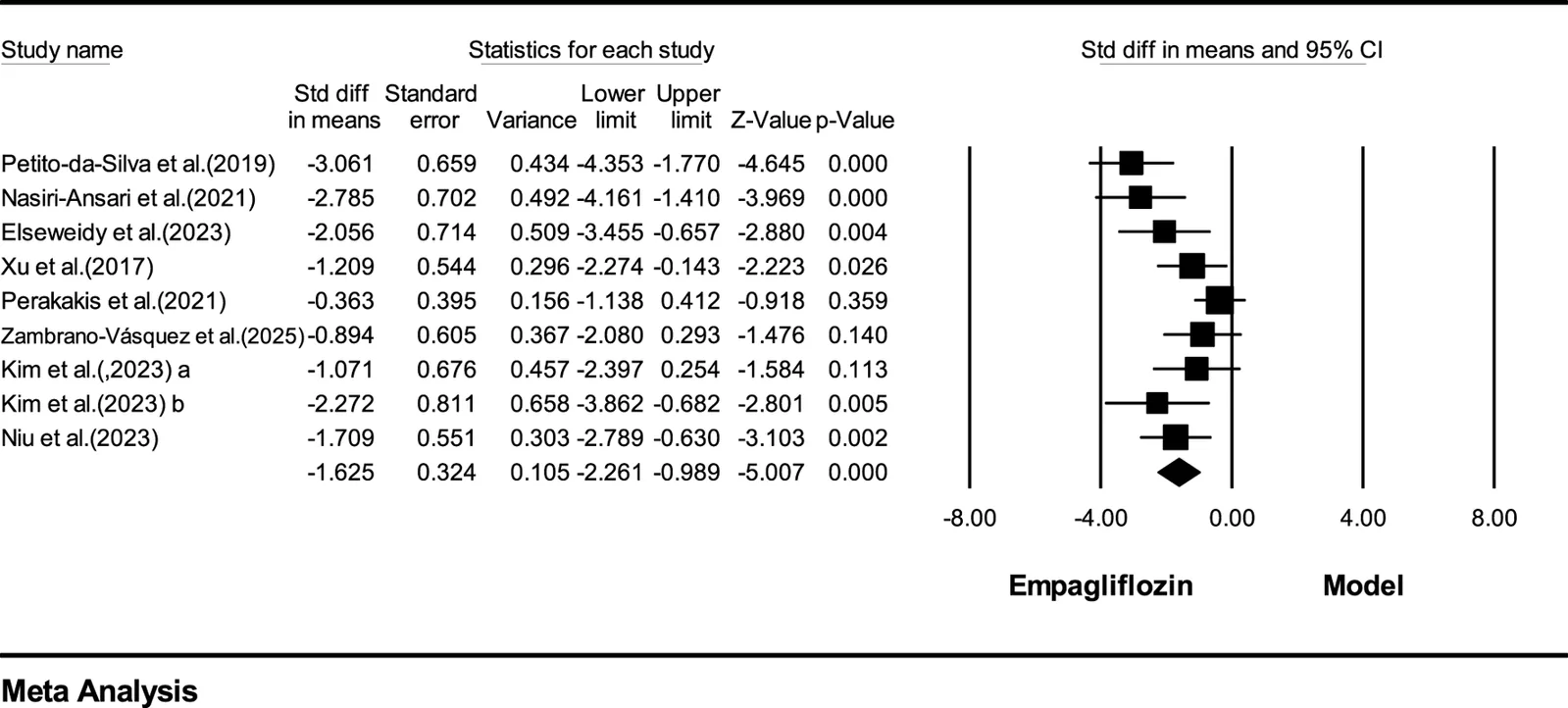
Serum cholesterol (mg/dl).

**FIGURE 9:**
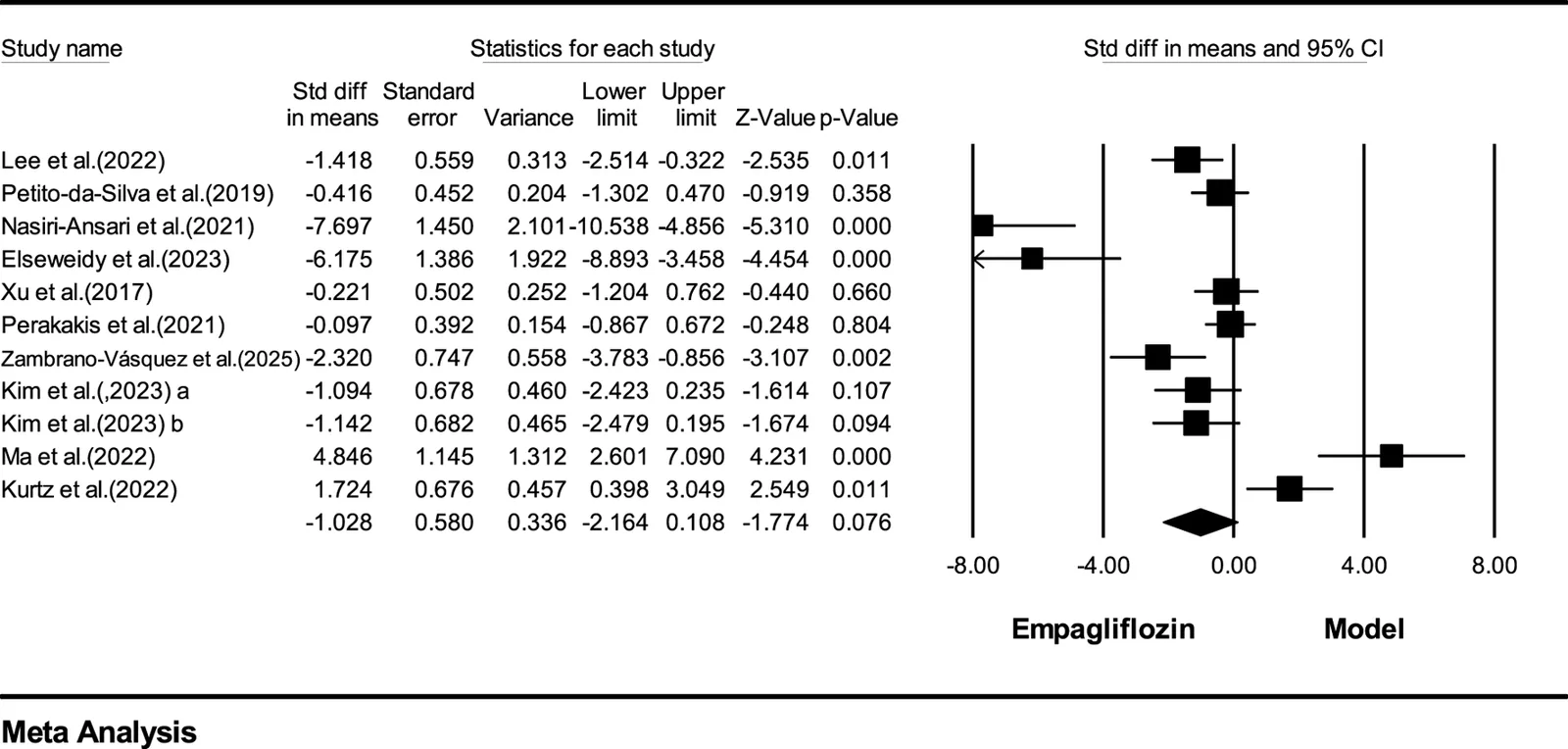
Serum TG (mg/dl).

The main markers of serum lipids, triglycerides and total cholesterol are also evaluated in our meta-analysis. Nine studies showed that empagliflozin groups had a significant reducing effect on serum cholesterol compared with the model groups [SMD = −1.625 (95% CI: -2.261 to −0.989); heterogeneity: I^2^ = 60.92%, P < 0.05] (Figure 8), regarding serum triglycerides eleven studies showed that empagliflozin groups had insignificant reducing effect on serum triglycerides compared with the model groups [SMD = −1.028 (95% CI: -2.164 to −0.108); heterogeneity: I^2^ = 88.3%, P > 0.05] (Figure 9).

### Effect of empagliflozin on fibrotic markers

**FIGURE 10:**
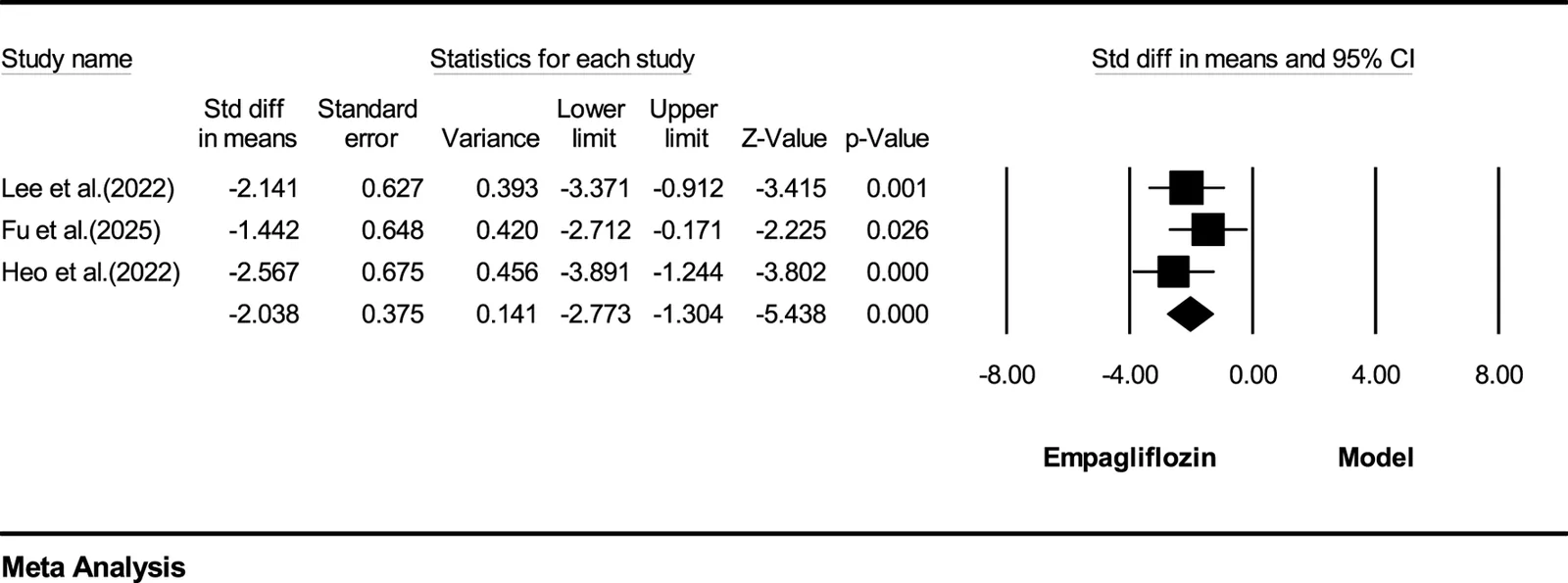
Fibrosis grade.

**FIGURE 11:**
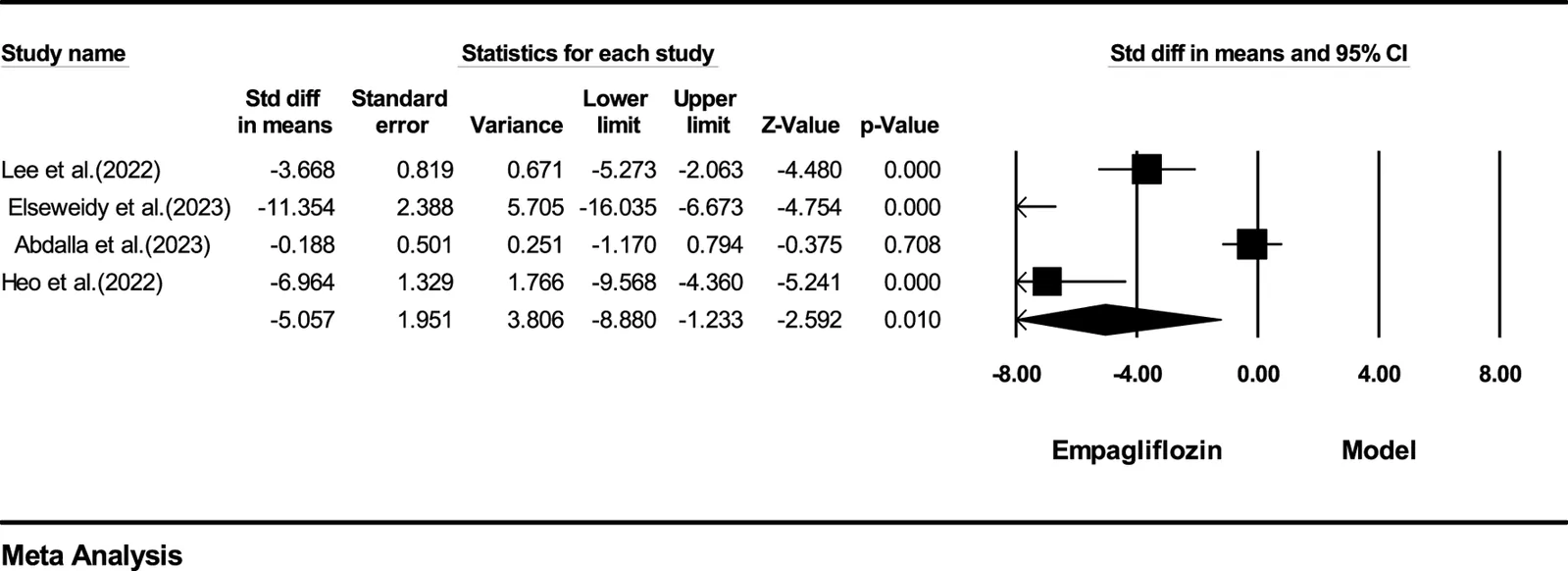
TGF-β.

**FIGURE 12:**
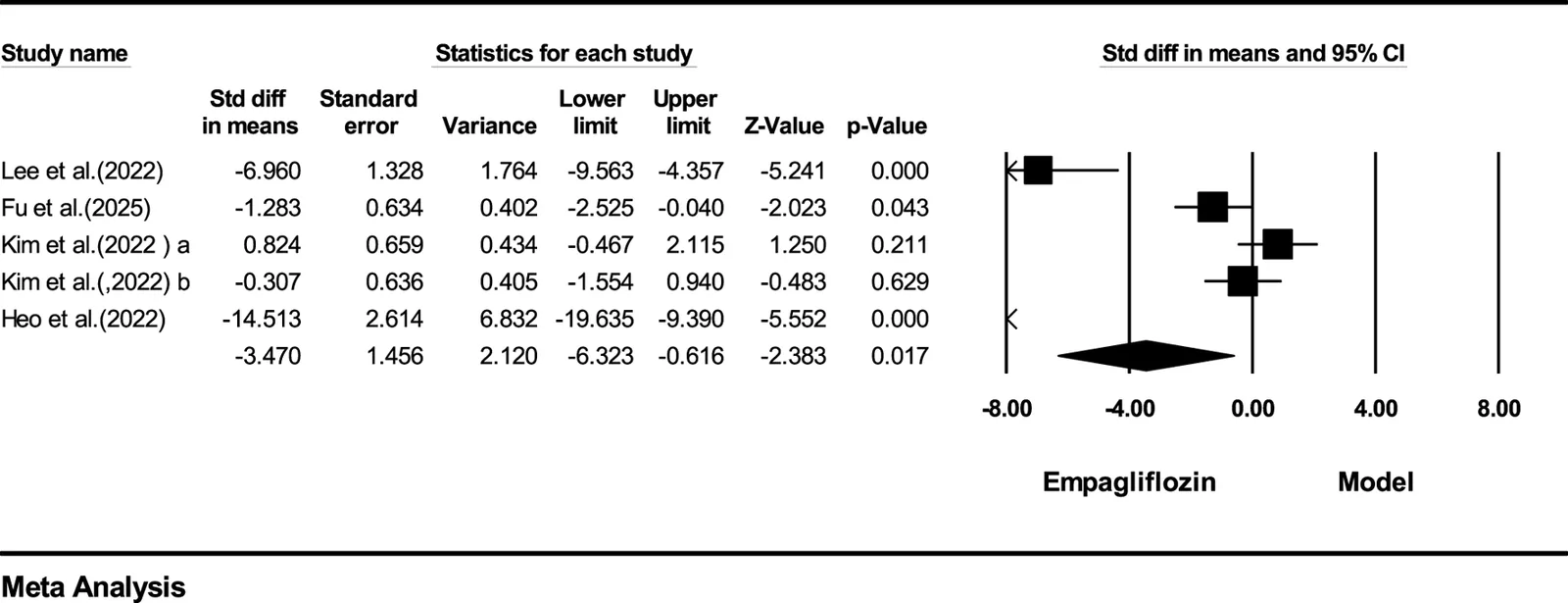
Hepatic COL1A1.

**FIGURE 13:**
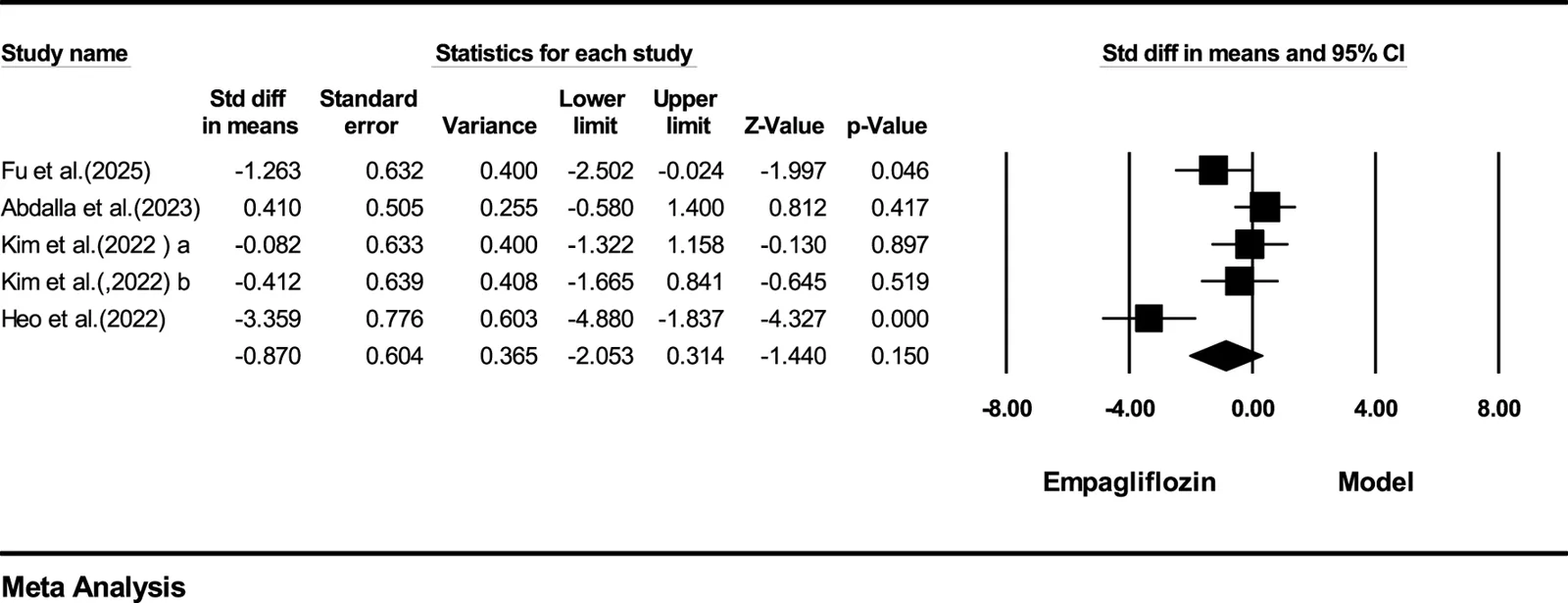
α-SMA.

Hepatic tissue fibrosis, which is a main marker for progression from MASLD to MASH (Khamis et al., 2025; Steinberg et al., 2025), is evaluated in our meta-analysis through studying the histopathological markers fibrosis grade and tissue markers including TGF-β,COL1A1 and α-SMA. Three studies assessed fibrosis grade, four studies assessed TGF-β relative expression and five studies assessed COL1A1 relative expression, all of them showed that empagliflozin groups had a significant reducing effect on fibrosis grade, TGF-β and COL1A1 compared with the model groups, for fibrosis grade [SMD = −2.038 (95% CI:-2.773 to −1.304); heterogeneity: I^2^= 00.00%, P <0.05] (Figure 10), for TGF-β [SMD = −5.057 (95% CI: -8.880 to −1.233); heterogeneity: I^2^ = 93.55%, P <0.05] (Figure 11), for COL1A1 [SMD = −3.47 (95% CI: -6.32 to −0.61); heterogeneity: I^2^ = 92.84%, P <0.05] (Figure 12). α-SMA is assessed in five studies, however, empagliflozin groups had insignificant reducing effect on α -SMA when compared to model groups [SMD = −0.870 (95% CI: -2.053 to 0.314); heterogeneity: I^2^ = 78.33%, P >0.05] (Figure 13).

### Effect of empagliflozin on liver enzymes

**FIGURE 14:**
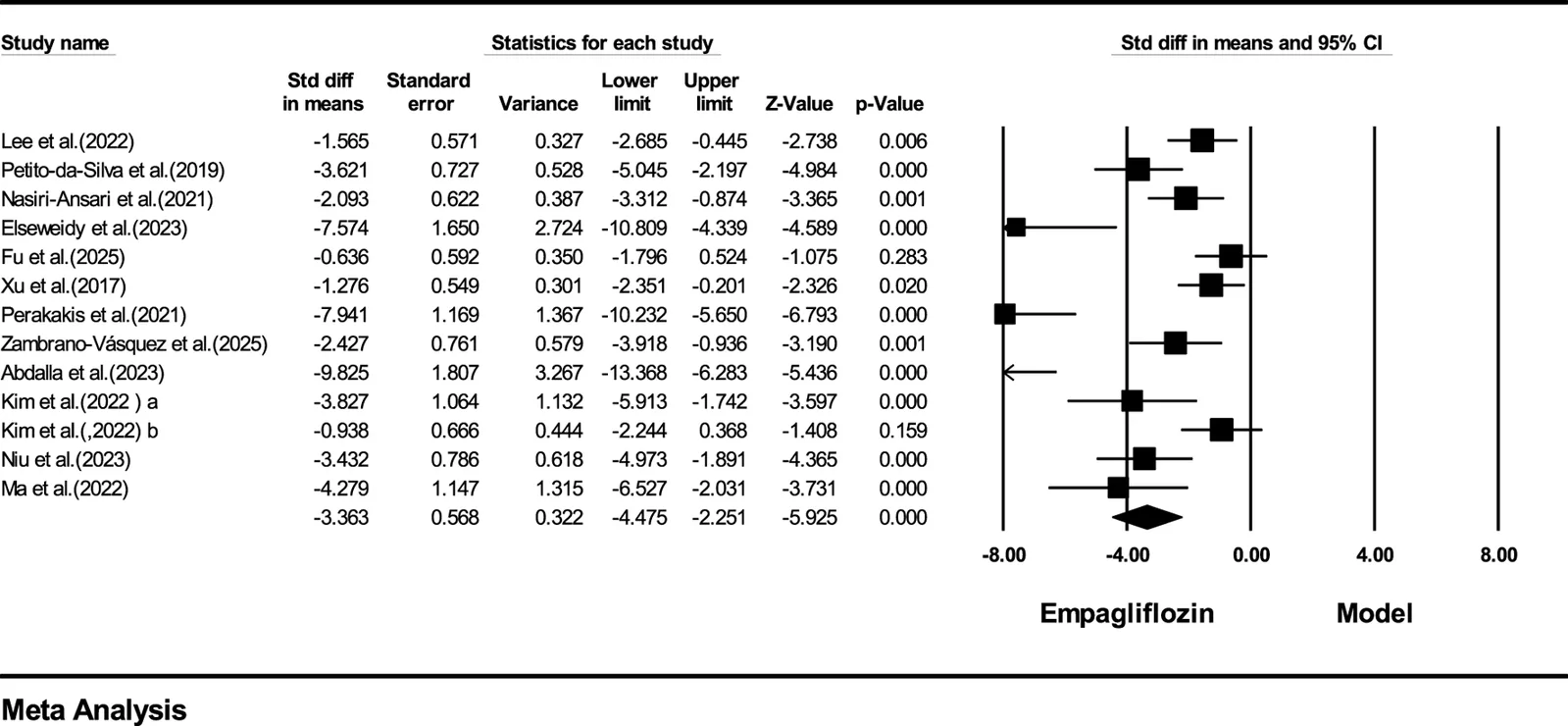
ALT.

**FIGURE 15:**
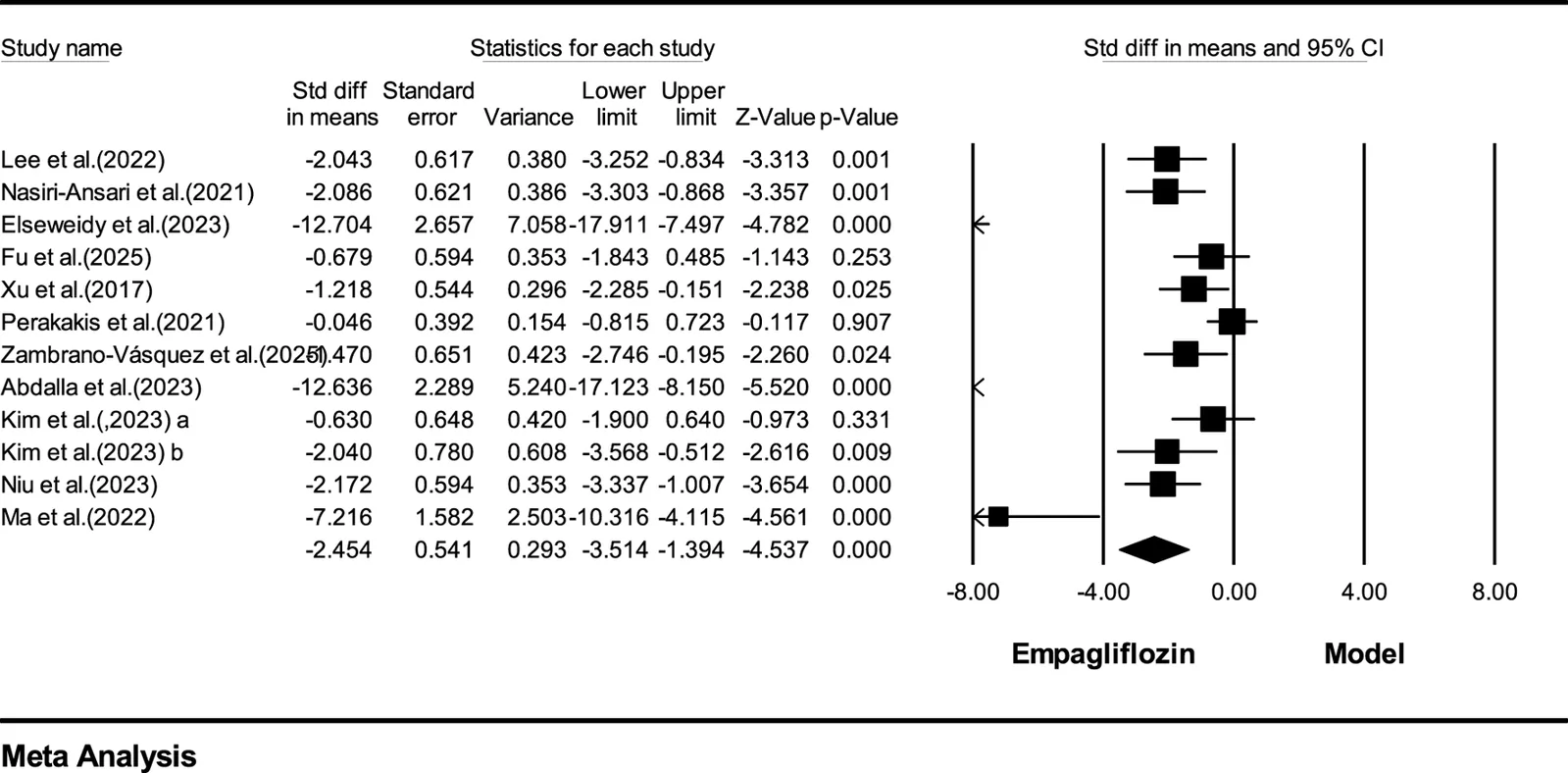
AST.

Liver enzymes ALT and AST which reflect hepatocyte injury that associates MASLD (Chen et al., 2025) are included in our meta-analysis. Thirteen studies assessed ALT and twelve studies assessed AST, both showed that empagliflozin groups had a significant reducing effect on serum levels of ALT and AST in empagliflozin groups when compared to model groups, for ALT [SMD = −3.365 (95% CI: -4.472 to −2.258); heterogeneity: I^2^= 84.85%, P <0.05] (Figure 14), and for AST [SMD = −2.454 (95% CI: -3.514 to −1.394); heterogeneity: I^2^ = 85.39%, P <0.05] (Figure 15).

## Secondary outcome measure

### Effect of empagliflozin on hepatic inflammatory markers

The degree of hepatic tissue inflammation is a main determinant of the speed of progress to fibrosis and cirrhosis (Kim et al., 2025). Many inflammatory markers are included in our meta-analysis including histopathological markers as lobular inflammation and molecular markers as TNF- α, IL-6 and F4/80.

**FIGURE 16:**
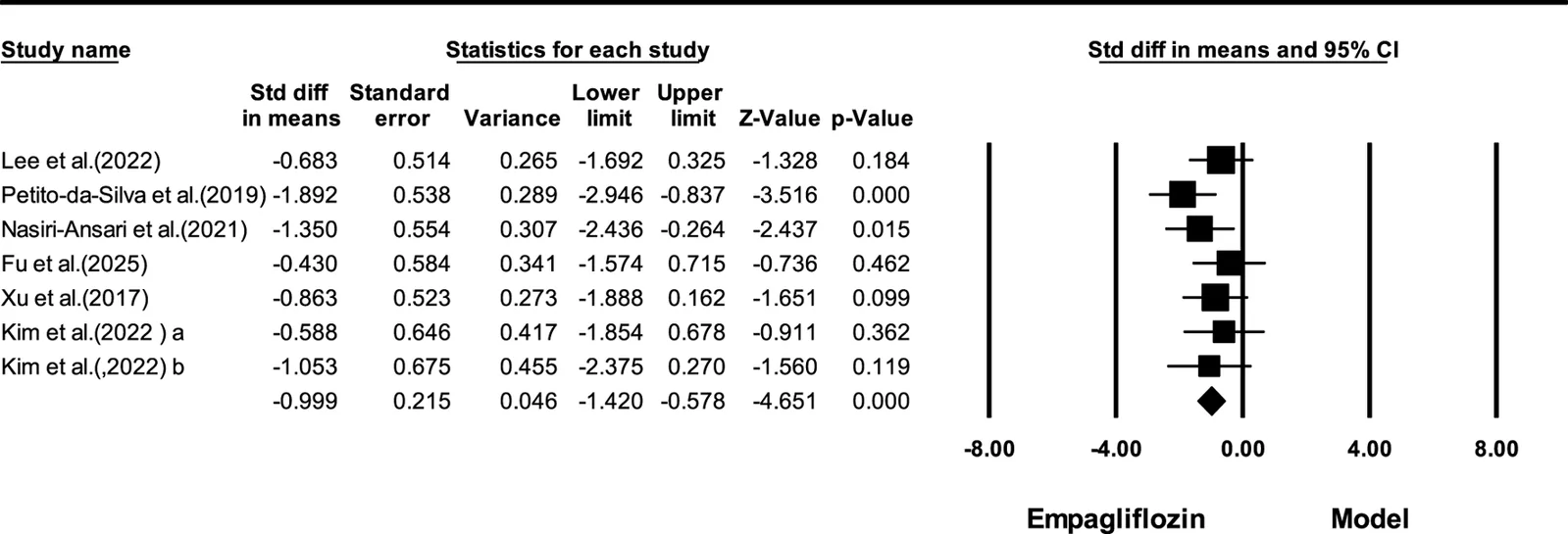
TNF- α.

**FIGURE 17:**
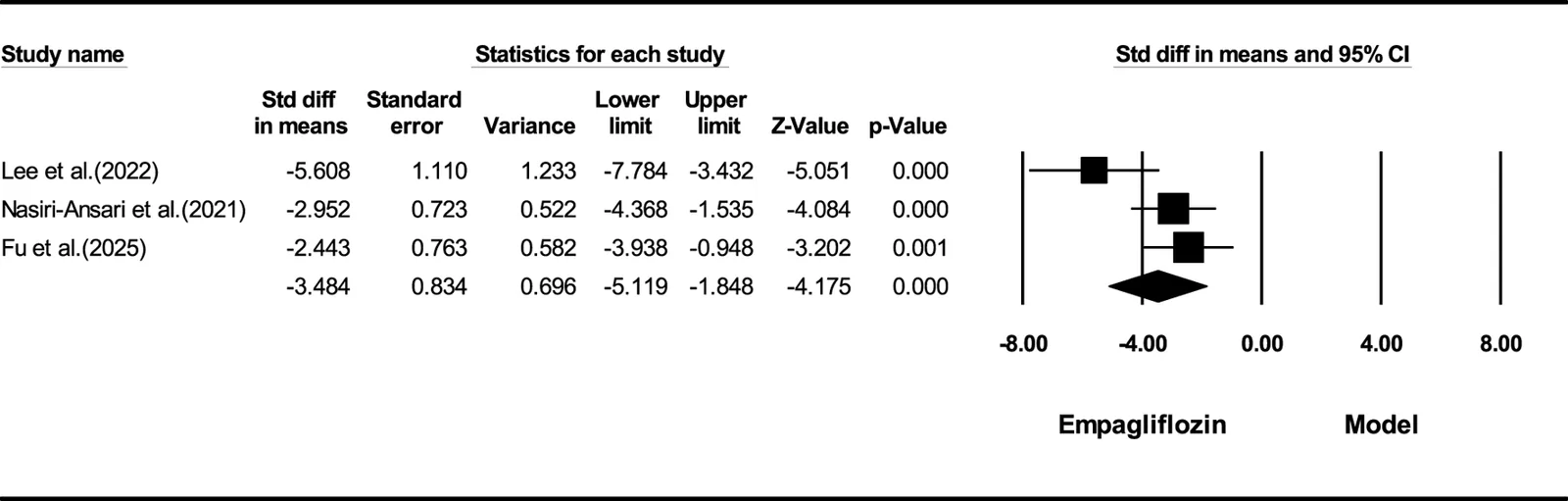
IL-6.

**FIGURE 18:**
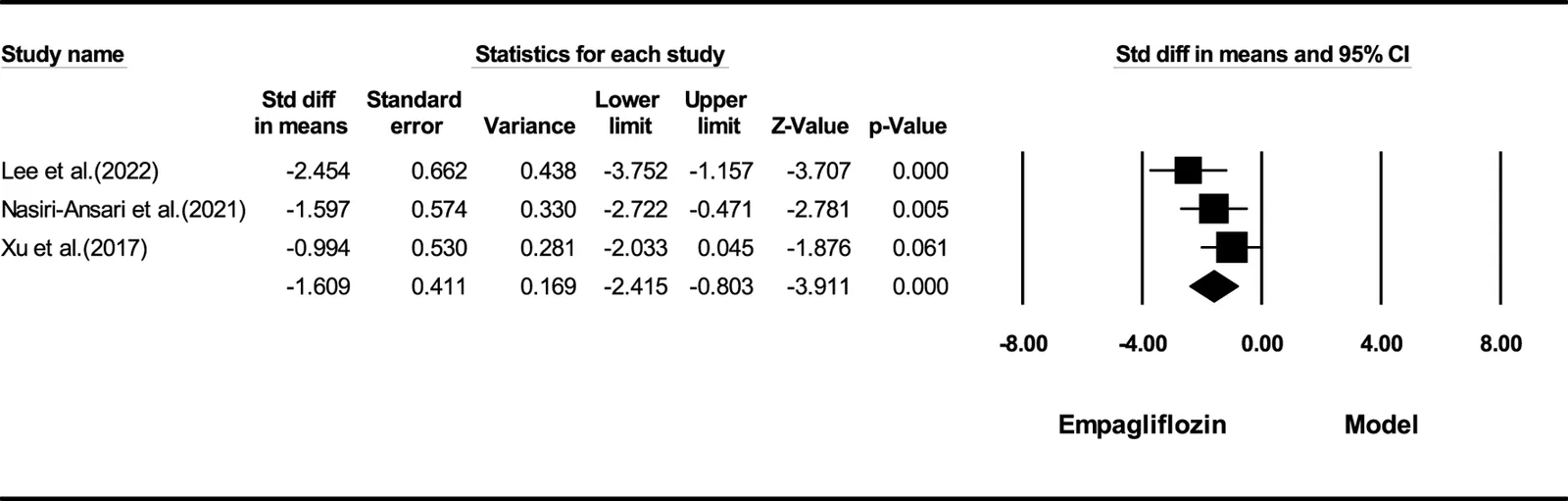
F4/80.

**FIGURE 19:**
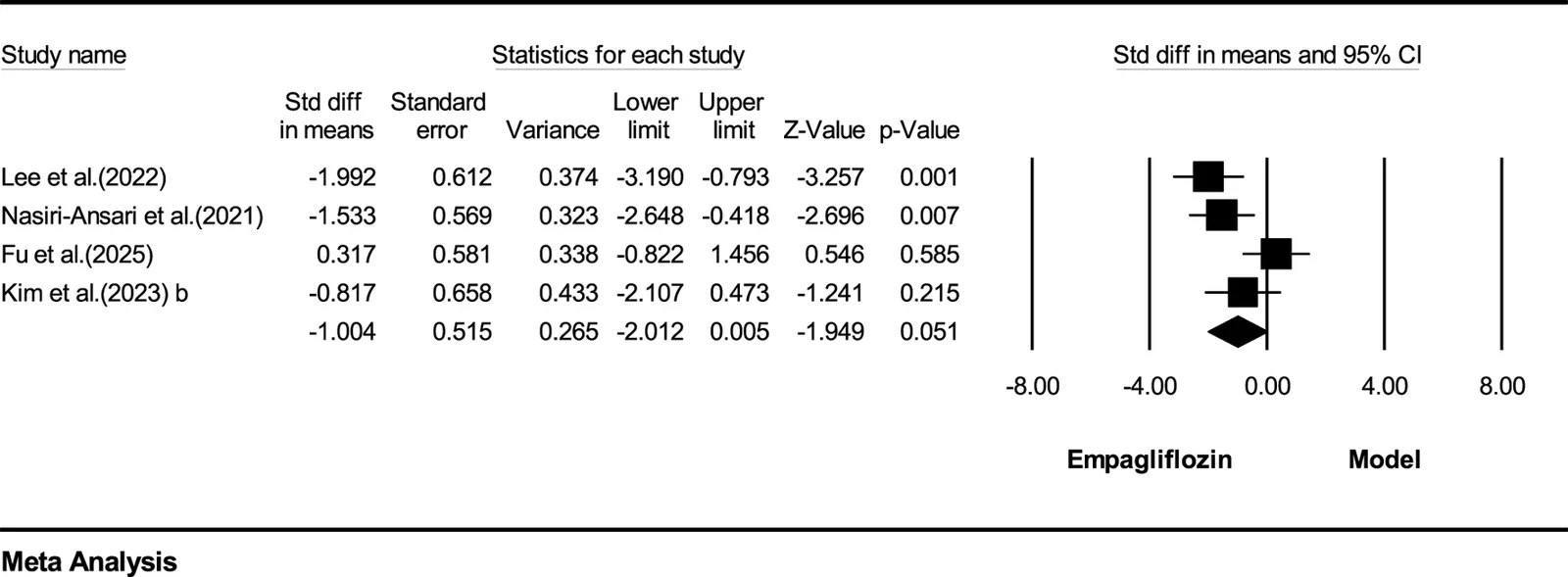
Lobular inflammation.

Seven studies assessed hepatic relative expression of TNF- α, three studies assessed the effect of empagliflozin on hepatic relative expression of IL-6 and three studies assessed hepatic relative expression of F4/80. All of the three markers were found to be significantly reduced in the empagliflozin groups compared to model groups, for TNF- α [SMD =-0.999 (95% CI: -1.420 to −0.578); heterogeneity: I^2^ = 0.00%, P <0.05] (Figure 16), for IL-6 [SMD=-3.484 (95% CI: -5.119 to −1.848); heterogeneity: I^2^ = 65.51%, P <0.05] (Figure 17) and for F4/80 [SMD = −1.609 (95% CI: -2.415 to −0.803); heterogeneity: I^2^= 32.57%, P <0.05] (Figure 18). Four studies assessed lobular inflammation which was insignificantly reduced in the empagliflozin group compared to model group [SMD=-1.004 (95% CI: -2.012 to −0.005); heterogeneity: I^2^ = 65.62%, P >0.05] (Figure 19).

### Effect of empagliflozin on hepatic oxidative stress markers

**FIGURE 20:**
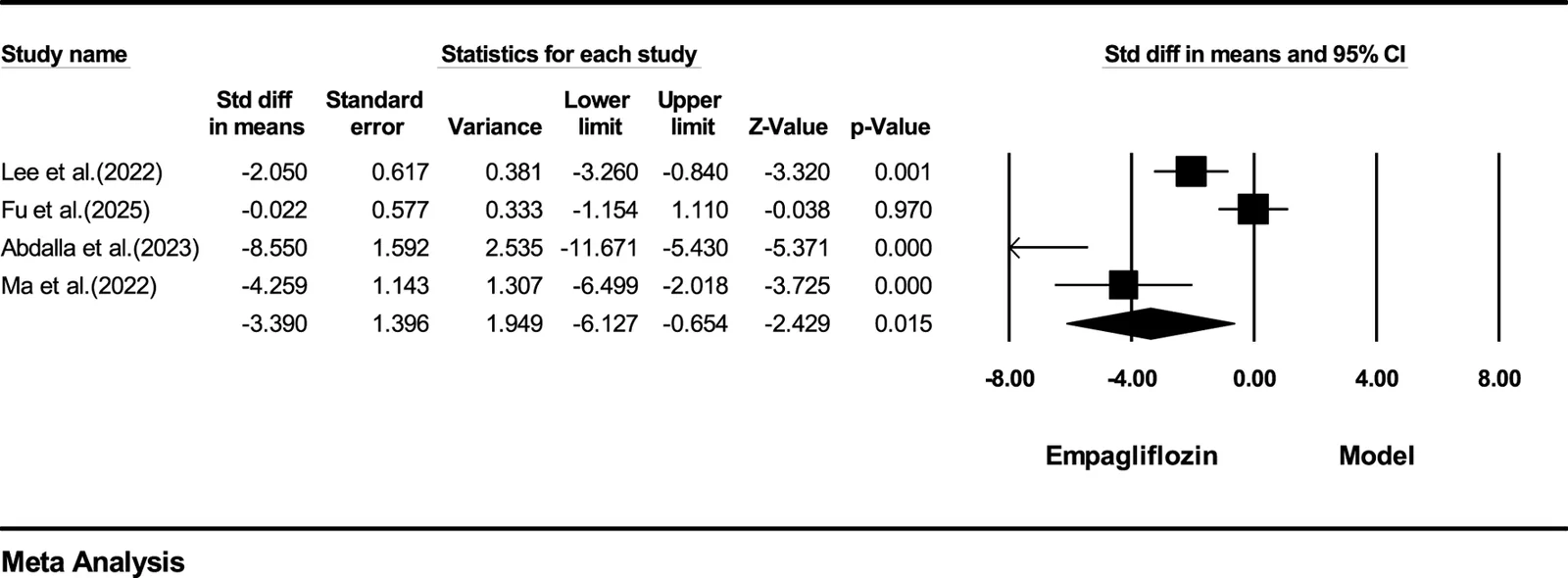
MDA.

**FIGURE 21:**
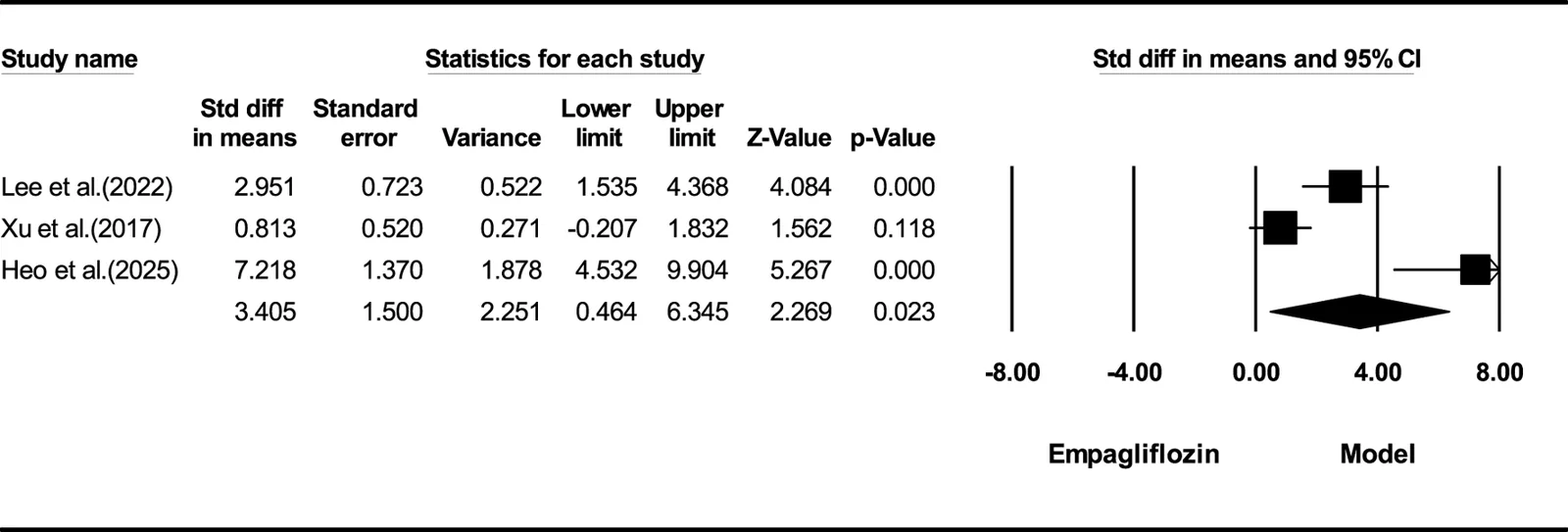
Catalase.

**FIGURE 22:**
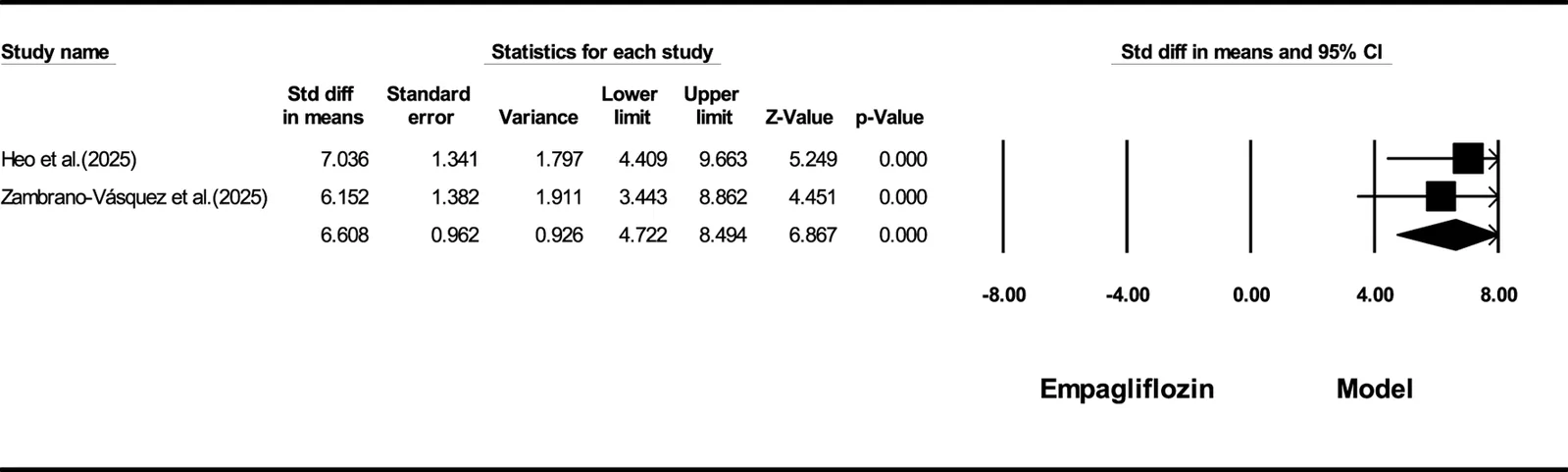
Nrf1.

Oxidative stress plays a central role in progression of MASD from simple steatosis to inflammation and fibrosis (Hu et al., 2025). Three main oxidative stress markers were included in our meta-analysis MDA, catalase and Nrf1. Results were towards reduction of the deleterious effects of the oxidative process, four studies showed that MDA was significantly reduced in the empagliflozin group compared to the model group [SMD = −3.390 (95% CI: -6.127 to −0.654); heterogeneity: I^2^ = 90.69%, P <0.05] (Figure 20). Moreover, three studies for catalase and two studies for Nrf1 showed that both anti-oxidant markers were significantly increased in the empagliflozin group when compared to the model group, for catalase [SMD= 3.405 (95% CI: 0.464 to 6.345); heterogeneity: I^2^ = 90.66%, P <0.05] (Figure 21) and for Nrf1 [SMD=6.608 (95% CI: 4.722 to 8.494); heterogeneity: I^2^ = 0.00%, P <0.05] (Figure 22).

### Effect of empagliflozin on body weight and liver weight

**FIGURE 23:**
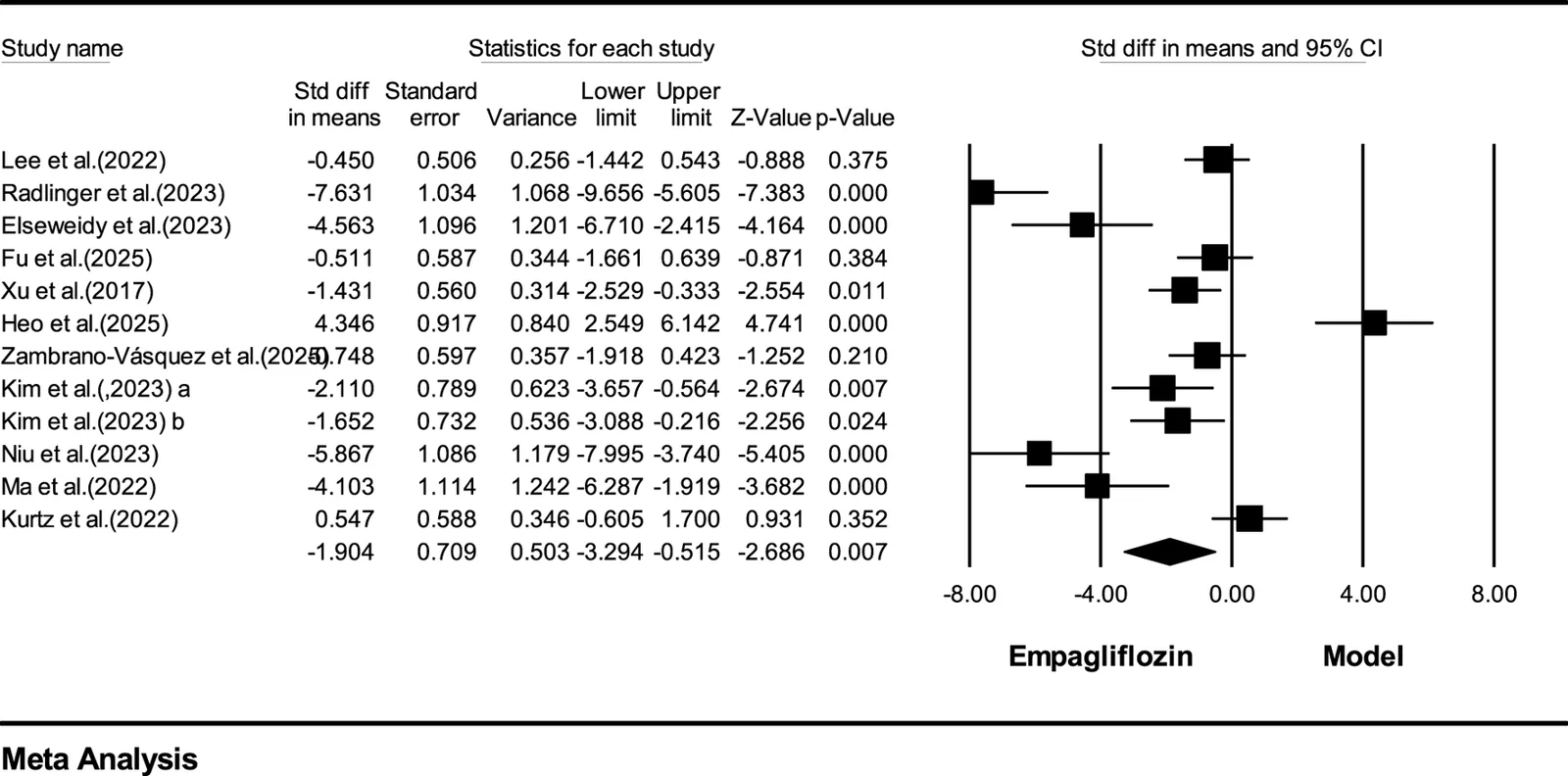
Body weight.

**FIGURE 24:**
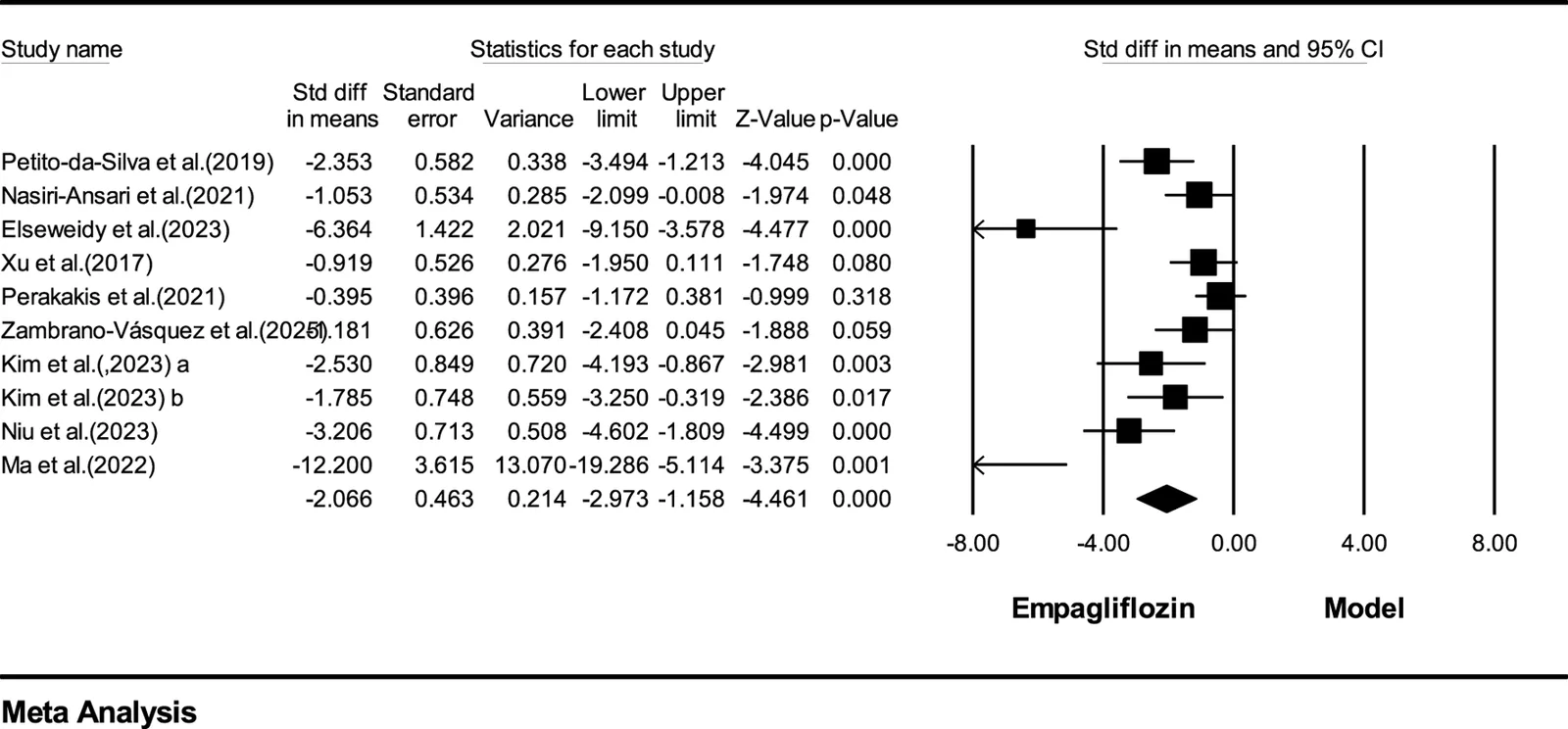
Liver weight.

One of the important non-glucose lowering effects of empagliflozin is its weight reducing effect (Kahl et al., 2022). Twelve studies reported the effect of empagliflozin on body weight in grams and ten studies reported the effect of empagliflozin on liver weight in grams. Body weight and liver weight were found to be significantly reduced in the empagliflozin groups when compared to the model groups, for body weight [SMD = −1.904 (95% CI: -3.294 to −0.515); heterogeneity: I^2^ = 91.19%, P < 0.05] (Figure 23) and for liver weight [SMD = −2.066 (95% CI: -2.973 to −1.158); heterogeneity: I^2^= 77.45%, P < 0.05] (Figure 24).

#### Effect of empagliflozin on glycemic markers: HOMA-IR, serum fasting insulin (µU/mL) and FBS (mg/dl)

**FIGURE 25:**
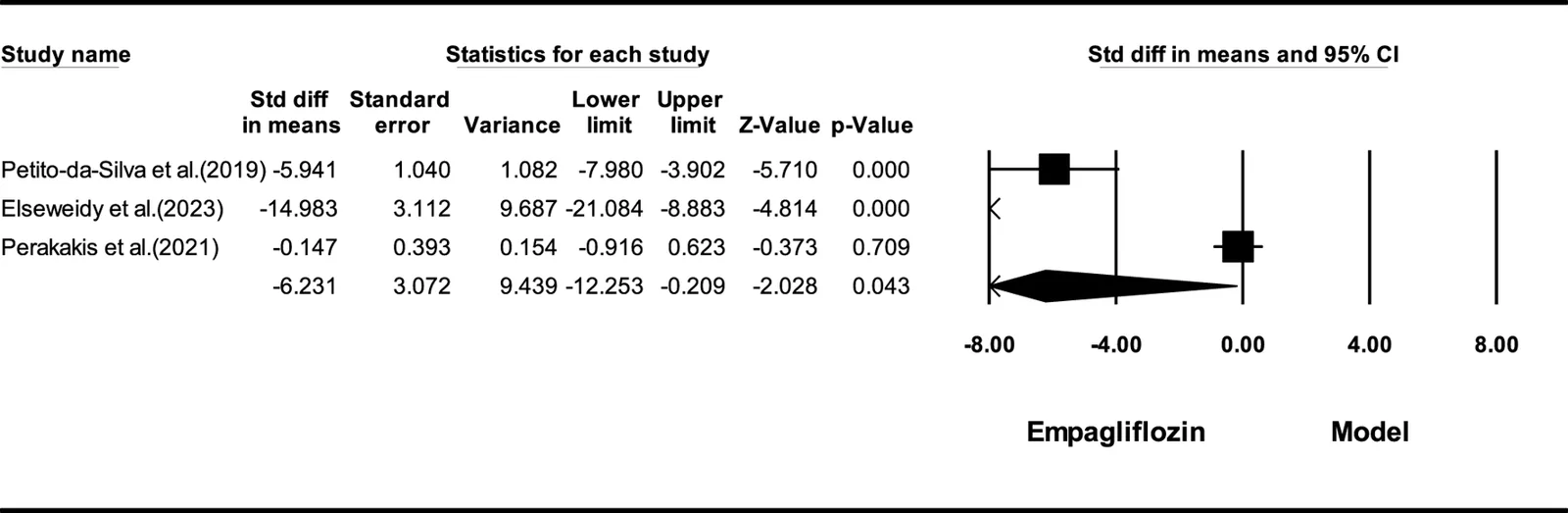
HOMA-IR.

**FIGURE 26:**
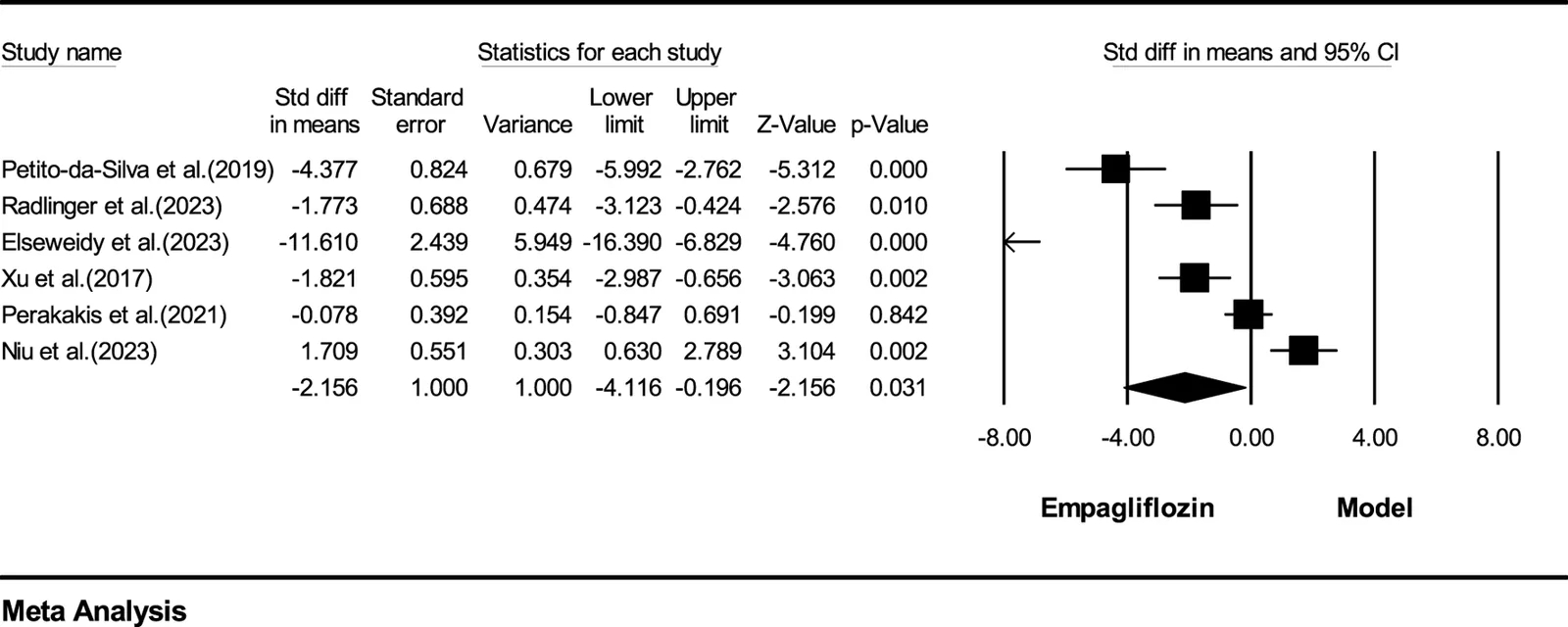
Serum fasting insulin.

**FIGURE 27:**
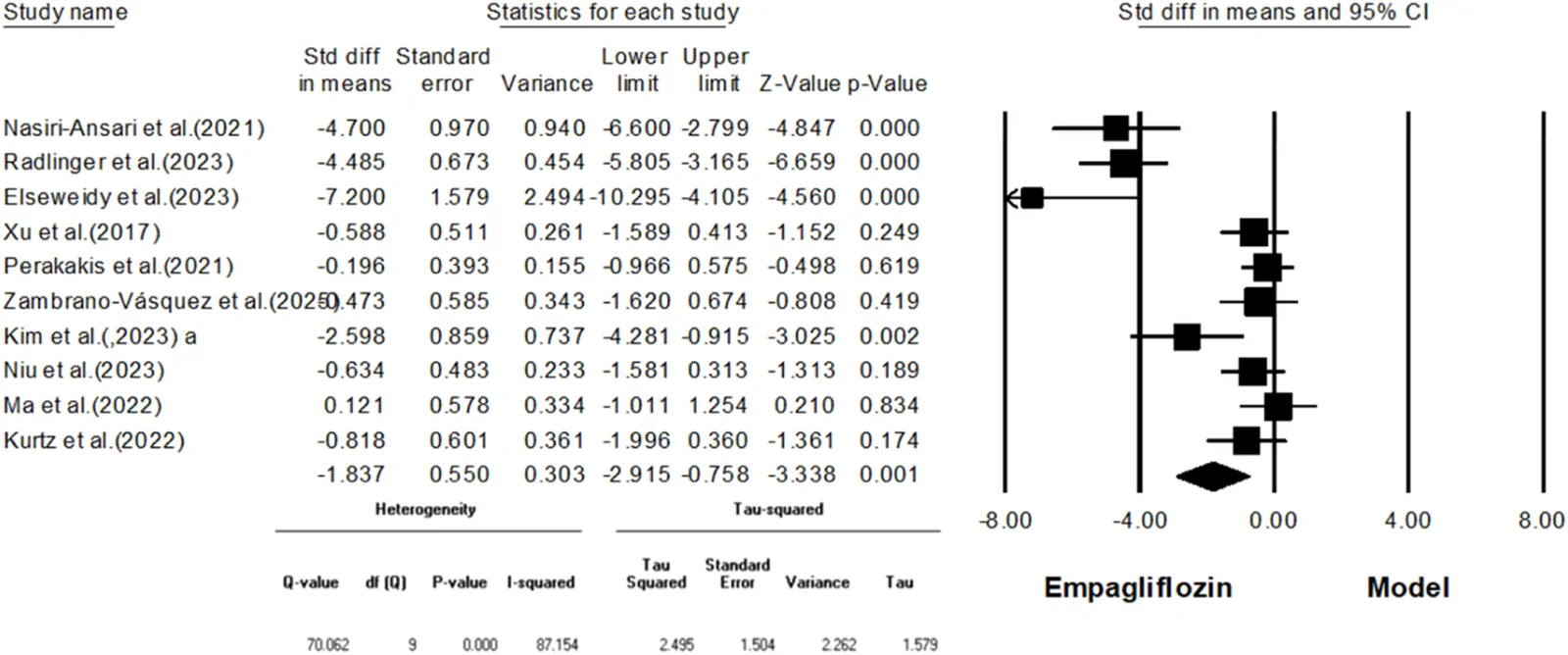
FBS.

Important clinical glycemic markers were also included in our preclinical met-analysis including HOMA-IR, serum insulin and FBS. Three studies assessed the effect of empagliflozin on HOMA-IR, six studies assessed the effect of empagliflozin on serum fasting insulin and ten studies assessed the effect of empagliflozin on FBS. The three main glycemic variables were found to be significantly reduced in the empagliflozin groups when compared to the model groups, for HOMA-IR [SMD = −6.231 (95% CI: -12.253 to −0.209); heterogeneity: I^2^ = 95.78, P <0.05] (Figure 25), for serum insulin [SMD = −2.156 (95% CI: -4.116 to −0.196); heterogeneity: I^2^ = 92.59, P >0.05] (Figure 26) and for FBS [SMD = −1.837 (95% CI: -2.915 to −0.758); heterogeneity: I^2^ = 87.15, P<0.05] (Figure 27).

### Subgroup analysis and sensitivity analysis

**FIGURE 28:**
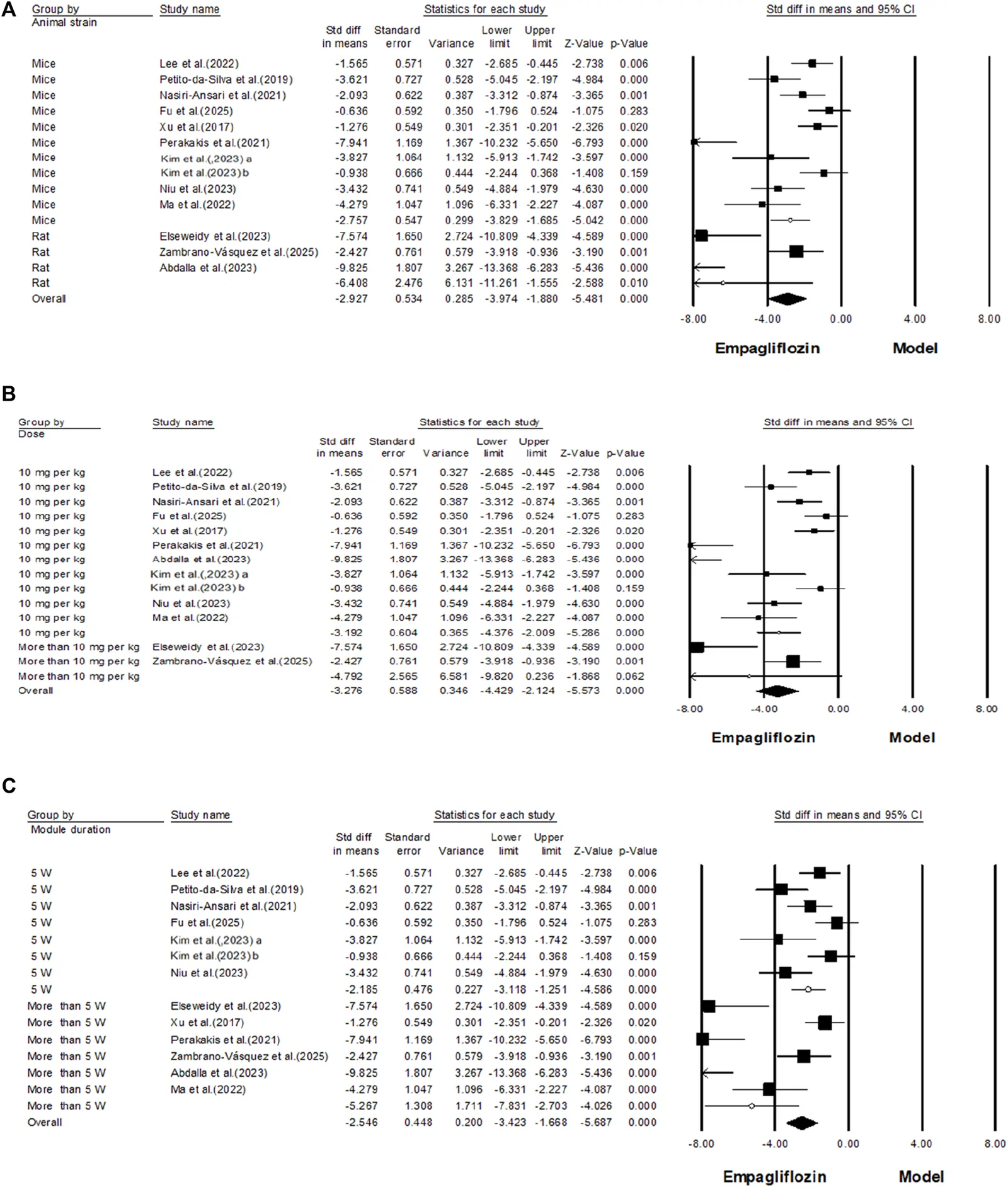
ALT subgroups. **(A)** ALT subgroups according to strain. **(B)** ALT subgroups according to dose. **(C)** ALT subgroups according to modelling duration.

**FIGURE 29:**
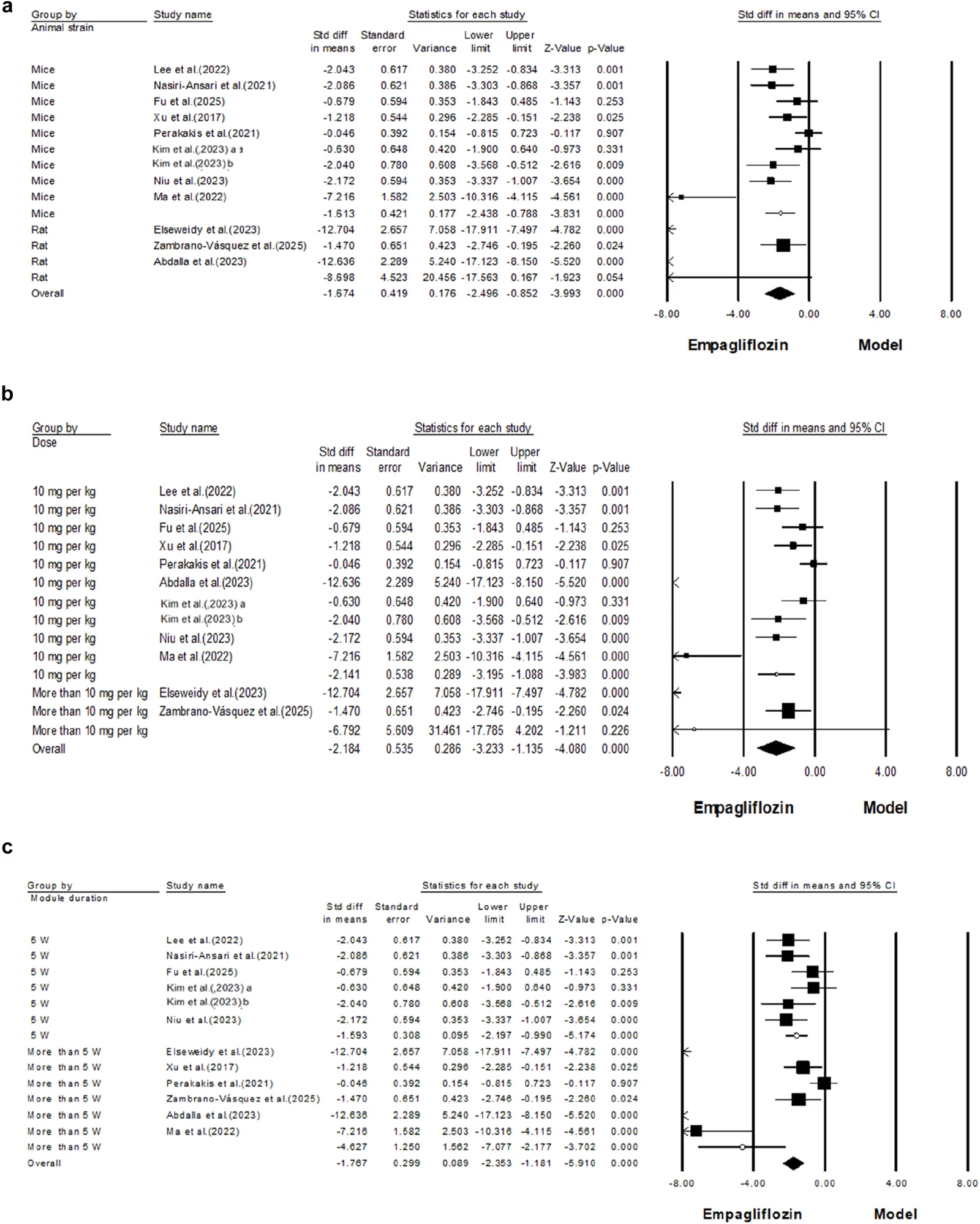
AST subgroups. **(a)** AST subgroups according to strain. **(b)** AST subgroups according to dose. **(c)** AST subgroups according to modelling duration.

**FIGURE 30:**
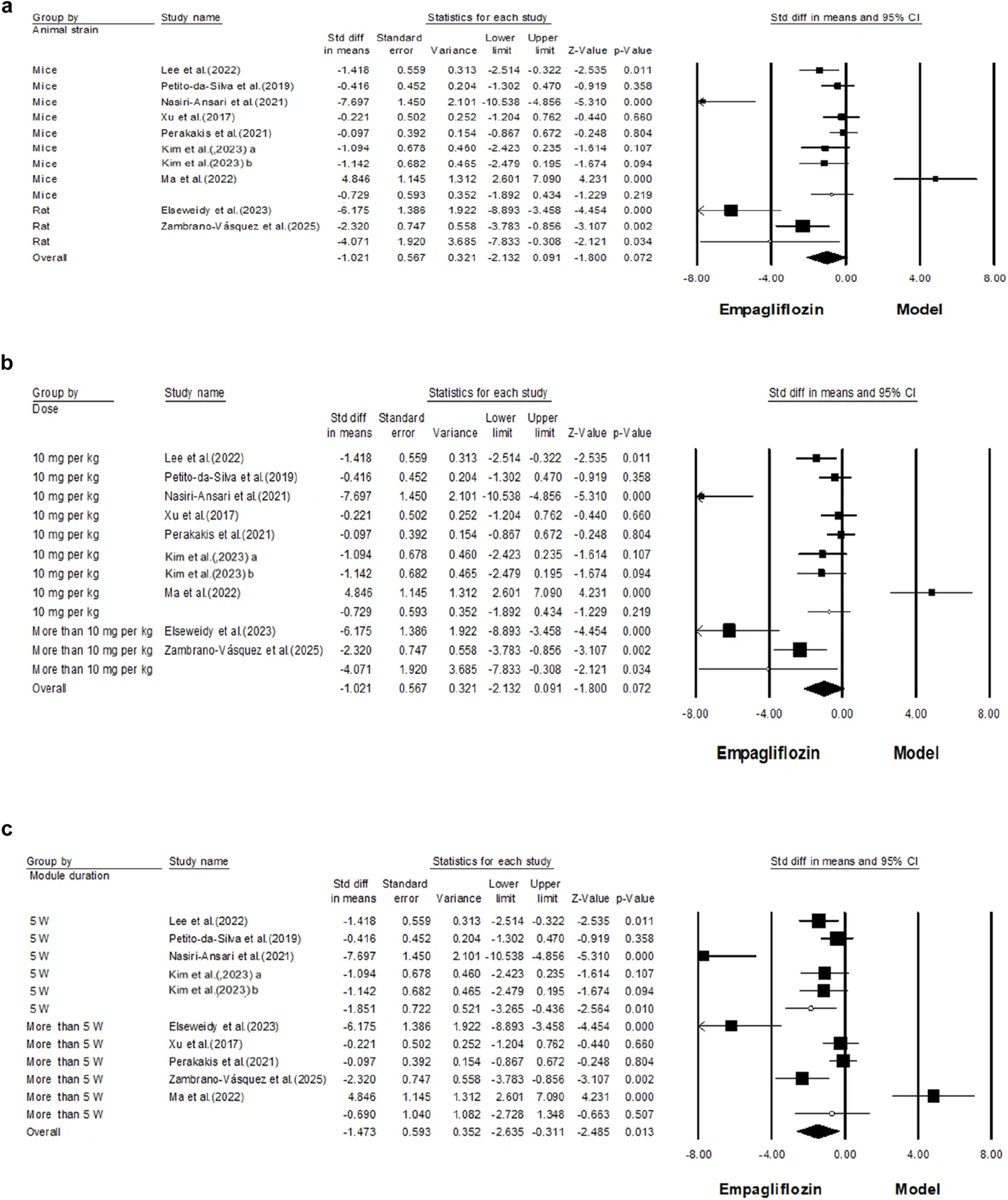
Serum triglycerides subgroups. **(a)** Serum TG subgroups according to strain. **(b)** Serum TG subgroups according to dose. **(c)** Serum TG subgroups according to modelling duration.

**FIGURE 31:**
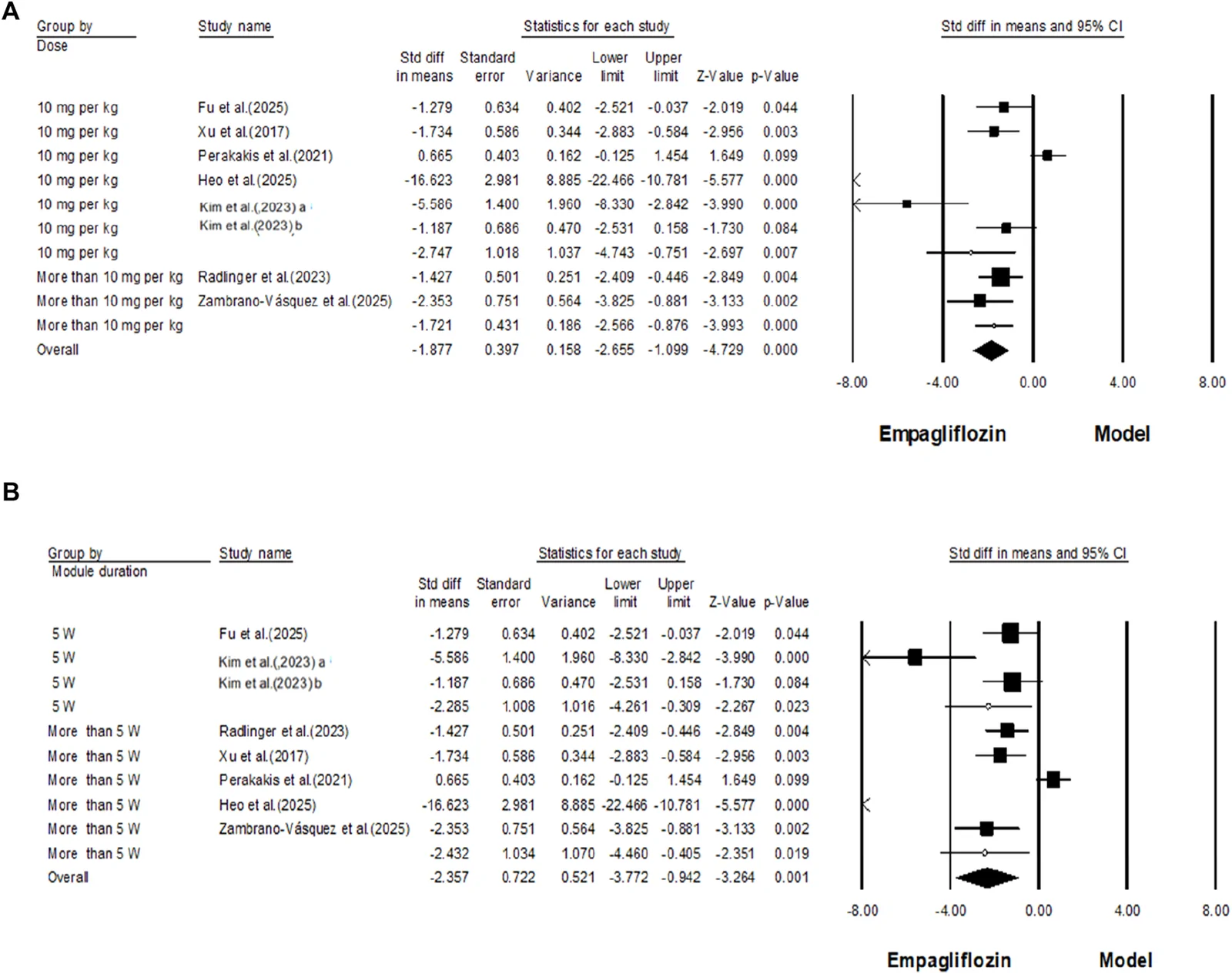
Hepatic triglycerides subgroups. **(A)** Hepatic TG subgroups according to dose. **(B)** Hepatic TG subgroups according to modelling duration.

Due to the high heterogeneity in the primary outcomes (I^2^ > 50%), a random-effects model was used for analysis, and we also conducted subgroup analyses by animal strain, modeling duration, and empagliflozin dose (Table 3 and Figures 28–31). Subgroup analyses showed that for ALT and hepatic triglycerides, high heterogeneity was most probably due to the empagliflozin dose, for AST was most probably due to modelling duration. Additionally, even after subgroup analysis, some subgroups still exhibited substantially high heterogeneity.

**TABLE 3:** subgroup analysis.

Variable	Subgroups	​	SMD [95% CI]	I^2^ (%)	*P* for heterogeneity
ALT	Strain	Rat	−6.4 (−11.26, −1.55)	89.63	<0.05
​	​	Mice	−2.75 (−3.82, −1.68)	82.58	<0.05
		Total	−2.92 (−3.97, −1.88)	84.85	<0.05
	Dose	10 mg/kg/day	−3.19 (−4.37, −2)	91.15	<0.05
	​	More than 10 mg/kg/day	−4.79 (−9.8, 0.2)	4.9	0.3
		Total	−3.27 (−4.42, −2.12)	88.52	<0.05
	Model duration	5 weeks	−2.18 (−3.11, −1.25)	70.17	<0.05
	​	More than 5 weeks	−5.26 (−7.83, -2.7)	90.25	<0.05
		Total	−2.54 (−3.42, −1.66)	84.85	<0.05
AST	Strain	Rat	−8.69 (−17.56, 0.16)	94.52	<0.05
​	​	Mice	−1.6 (−2.43, -0.78)	75.28	<0.05
		Total	−1.67 (−2.49, −0.85)	85.39	<0.05
	Dose	10 mg/kg/day	−2.14 (−3.19, −1.08)	84.21	<0.05
	​	More than 10 mg/kg/day	−6.79 (−17.78, 4.2)	94	<0.05
		Total	−2.18 (−3.23, −1.13)	85.39	<0.05
	Model duration	5 weeks	−1.59 (−2.19, 0.99)	28.74	0.21
	​	More than 5 weeks	−4.62 (−7, −2.1)	92.53	<0.05
		Total	−1.76 (−2.35, −1.18)	85.39	<0.05
Serum TG	Strain	Rat	−4.07 (−7.83, 0.3)	86.57	<0.05
​	​	Mice	0.72 (-1.89, 0.43)	83.3	<0.05
		Total	−1.02 (−2.13, 0.09)	87.6	<0.05
	Dose	10 mg/kg/day	−0.72 (−1.89, 0.43)	86.57	<0.05
	​	More than 10 mg/kg/day	−4.07 (−7.83, −0.3)	83.31	<0.05
		Total	−1.02 (−2.13, 0.09)	87.67	<0.05
	Model duration	5 weeks	−1.85 (−3.26, 0.43)	82.81	<0.05
	​	More than 5 weeks	−0.69 (−2.72, 1.34)	91.23	<0.05
		Total	−1.47 (−2.63, 0.31)	87.67	<0.05
Hepatic TG	Dose	10 mg/kg/day	−2.74 (−4.74, 0.75)	91.15	<0.05
​	​	More than 10 mg/kg/day	−1.72 (−2.56, 0.87)	4.95	0.3
		Total	−1.87 (−2.65, 1.09)	88.52	<0.05
	Model duration	5 weeks	−2.28 (−4.26, 0.3)	77.01	<0.05
	​	More than 5 weeks	−2.43 (−4.46, 0.4)	91.95	<0.05
		Total	−2.35 (−3.77, 0.94)	88.52	<0.05

We performed sensitivity analysis by removing one study at a time; there was no change in the significance of results for most of the primary outcomes, including hepatic TG, hepatic cholesterol, NAS score, oil red area, fibrosis grade, α-SMA, ALT, and AST. Only for hepatocyte ballooning, removal of Zambrano-Vásquez et al. (2025), for serum TG, removal of Ma et al. (2022) and Kurtz et al. (2022) and for TGF-β, removal of Lee et al. (2022), Elseweidy et al. (2024) and Abdalla et al. (2023) caused minor changes in the significance, so these results must be considered with caution.

### Publication bias

**FIGURE 32:**
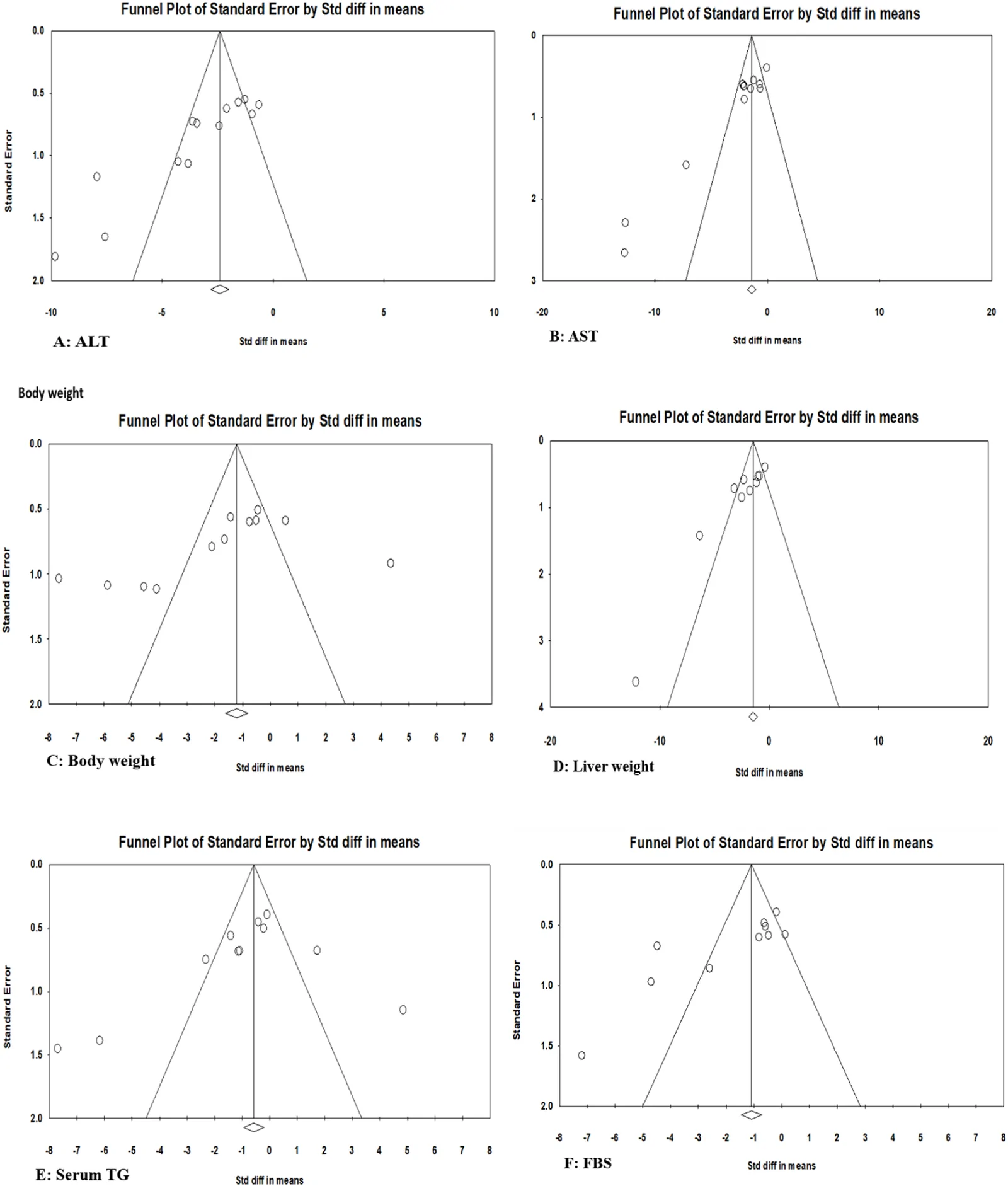
Funnel plots for **(A)** ALT, **(B)** AST, **(C)** Body weight, **(D)** Liver weight, **(E)** Serum TG, and **(F)** FBS.

- Publication bias was done for outcome indicators in 10 or more studies, first we performed funnel plots followed by Egger’s regression (Figure 32).
- Egger’s test indicated that publication bias was statistically significant for FBS, AST, ALT, and liver weight (P < 0.05) but not for triglycerides or body weight.
- Finally we performed trim and fill analysis to assess the impact of this publication bias. Trim and fill detected only one missing study that did not affect the stability of the results**.**

## Discussion

Empagliflozin has gained increasing attention as a pleiotropic metabolic regulator extending beyond its glucose-lowering effects. Growing evidence indicates that empagliflozin favorably modulates key pathogenic processes relevant to MASLD. Specifically, empagliflozin attenuates hepatic oxidative stress, enhances endogenous antioxidant capacity, preserves mitochondrial integrity and exerts strong anti-inflammatory effects (Comella et al., 2025). These diverse effects collectively mitigate hepatocellular injury and reduce the risk of developing steatohepatitis with the subsequent liver cirrhosis and hepatocellular carcinoma (Smati et al., 2024).

Importantly, much of the current clinical evidence is derived from observational human studies that rely on surrogate biochemical, imaging endpoints and noninvasive markers. Although clinically informative, they provide limited mechanistic resolution. In contrast, animal models offer a robust experimental framework to interrogate causality, enabling controlled manipulation of metabolic stressors, direct tissue sampling and detailed molecular analyses. Even more, animal studies can help in histopathological score and evaluation using validating scoring systems such as NAS, which remains the gold standard for MASLD evaluation. These advantages allow precise characterization of anti-oxidative and anti-inflammatory pathways and facilitate a mechanistic interpretation that is not feasible in human studies. Accordingly, integration of animal-based mechanistic evidence with clinical observations is essential for clarifying the beneficial hepatoprotective actions of empagliflozin in MASLD and for strengthening its translational relevance.

## Integration of the Multiple-Hit Theory of MASLD and Empagliflozin’s Hepatoprotective Actions

The pathogenesis of MASLD is currently explained by the multiple-hit hypothesis, which proposes that hepatic steatosis, inflammation, oxidative stress and fibrogenesis arise from the parallel and synergistic interaction of several metabolic and molecular insults rather than a single linear process (Devasia et al., 2025).

The main drivers are obesity and insulin resistance, which promote excessive free fatty acid flux to the liver and stimulate *de novo* lipogenesis, leading to pathological lipid accumulation within hepatocytes (Friedman et al., 2018). The resulting lipotoxic imbalance induces mitochondrial dysfunction and excessive reactive oxygen species generation, thereby amplifying oxidative stress and triggering inflammatory signaling pathways (Wang et al., 2025). Persistent activation of innate immune responses, characterized by increased production of pro-inflammatory cytokines such as TNF-α and IL-6, further exacerbates hepatocellular injury and facilitates progression from simple steatosis to steatohepatitis (Duan et al., 2022). In parallel, sustained oxidative and inflammatory stress activates hepatic stellate cells through profibrotic mediators such as TGF-β, COL1A1, and α-SMA, causing extracellular matrix deposition and fibrosis (Solleiro-Villavicencio et al., 2025).

In addition to the pivotal role of the multiple-hit hypothesis, the pathophysiology of MASLD extends to involve other organs related to the liver-gut axis, especially the spleen. In the context of metabolic stress, the spleen becomes a significant source of pro-inflammatory cytokines, such as TNF-α and IL-6, which are transported directly to the liver through portal circulation. This constant influx of inflammatory mediators exacerbates hepatic insulin resistance and accelerates the transition to more severe inflammation and fibrosis (Tarantino et al., 2021). Furthermore, the spleen acts as a reservoir for specialized immune cells, including certain macrophages and T-cells, which can migrate to the liver and intensify the local immune response. As the disease progresses, the spleen may also undergo structural changes like splenomegaly, which further disrupts systemic lipid metabolism and iron homeostasis, creating a feedback loop that fuels the chronic inflammatory state central to MASLD pathogenesis (Zhang et al., 2022).

**FIGURE 33:**
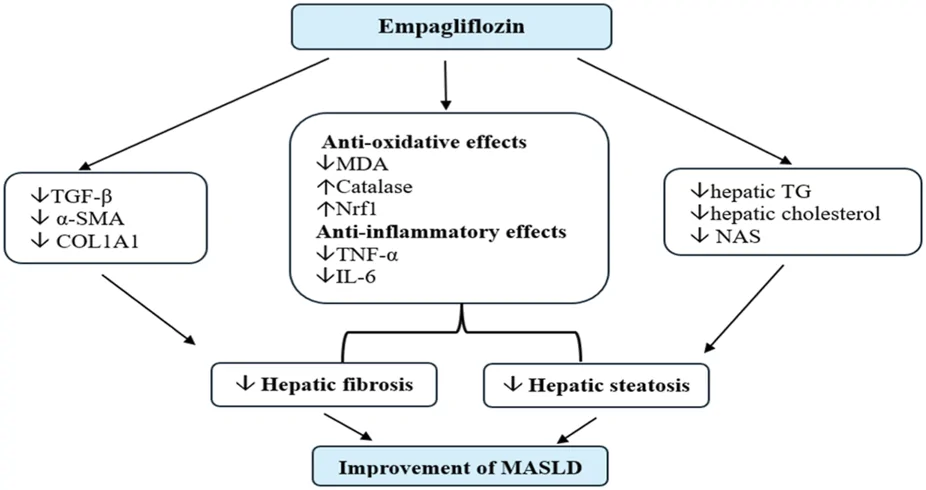
Summary of the protective effects of empagliflozin against MASLD.

Our meta-analysis shows a multifaceted pharmacodynamic profile of empagliflozin that “hits” many of the core pathogenic nodes of the “multiple-hit hypothesis of MASLD” in several ways, which helps to mitigate its metabolic and clinical consequences. We developed a schematic mechanistic diagram to illustrate these effects (Figure 33).

First, regarding our primary outcomes, empagliflozin modulated key lipid-related parameters to correct this metabolic imbalance. It significantly reduced hepatic triglycerides and cholesterol thus slowing down the process of intrahepatic lipid accumulation. Moreover, empagliflozin significantly reduced serum cholesterol and serum TG. These findings are supported by several clinical studies (Abdelgani et al., 2024; El-Kassas et al., 2025; Suhardi et al., 2025).

Second, the anti-fibrotic effects observed in preclinical MASLD models are of particular translational relevance, given that the fibrosis stage represents the strongest histological predictor of liver-related and all-cause mortality in patients with non-alcoholic steatohepatitis. While clinical data on SGLT2 inhibitors in MASLD have largely focused on improvements in hepatic steatosis, aminotransferases, and metabolic parameters, emerging evidence suggests potential benefits on fibrosis-related surrogates (Erfanifar et al., 2025). The present meta-analysis provides mechanistic support for these observations by demonstrating that empagliflozin consistently suppresses key fibrogenic pathways, including significant downregulation of transforming growth factor-β and COL1A1 alongside reductions in hepatic inflammation and oxidative stress, the two major upstream drivers of hepatic stellate cell activation (Fu et al., 2025). These findings suggest a possible protective effect of empagliflozin against hepatic steatosis and fibrosis.

Third, our meta-analysis also revealed that empagliflozin regulates blood glucose by other mechanisms extending beyond its established role in inhibiting glucose reabsorption in the proximal tubules. Notably, insulin resistance, which is a core feature of metabolic syndrome, also served as a crucial pathological basis for diseases such as MASLD (Yang SM. et al., 2023). Existing studies have indicated that empagliflozin exhibits certain potential in improving insulin resistance, and its mechanism of action is closely associated with regulating metabolic pathways, inhibiting inflammatory responses, and alleviating oxidative stress (Li et al., 2024). Moreover, empagliflozin significantly improved other main glycemic indices such as serum insulin and FBS.

Fourth, disturbed hepatic and systemic metabolism in MASD is thought to be mainly due to profound inflammatory response and oxidative stress accompanying hepatocyte lipotoxicity (Solleiro-Villavicencio et al., 2025). Our meta-analysis showed that empagliflozin attacks both mechanisms, where it significantly reduced the hepatic expression of the main inflammatory markers TNF-α and IL6 (Jojima et al., 2016). Also, it regulated the oxidative process where it significantly downregulated the hepatic expression of MDA and upregulated the expression of catalase and Nrf1. Fifth, impaired liver enzymes are a key indicator of hepatocellular damage in MASLD (Grob et al., 2023). Our study showed that empagliflozin had the effect of reducing liver enzymes. 13 studies on ALT and 12 studies on AST showed that empagliflozin significantly reduced this marker. This reduction indicates less hepatocyte necrosis and inflammation. The effect size for ALT was larger than for AST. This is not the same in clinical studies, where many studies showed a significant reduction in liver enzymes (Kuchay et al., 2018; Sattar et al., 2018), while others showed no significant differences (Chehrehgosha et al., 2021). Such notable effects of empagliflozin on hepatic enzymes, hepatic inflammation, and oxidative stress suggest a possible mitigating effect of empagliflozin against the pathophysiology of MASLD.

Sixth, our results showed a significant decrease in body weight, which is mostly attributed to decreased steatosis, diuretic effects, natriuretic effects, and improved insulin sensitivity. This comes in harmony with the results of most, but not all, previous clinical studies (Danpanichkul et al., 2025). Also, the studies revealed a significant decrease in liver weight, which is mostly attributed to lower intrahepatic steatosis**.**

**FIGURE 34:**
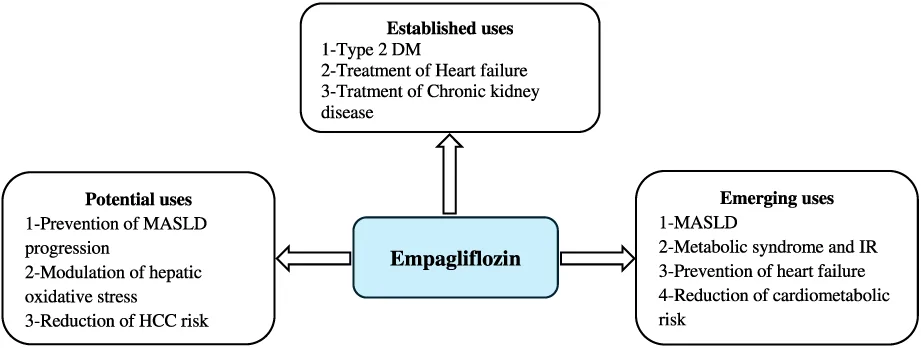
Scope of clinical uses of empagliflozin.

In summary, Empagliflozin was associated with the reduction of hepatic steatosis, attenuation of chronic inflammatory signaling, and suppression of fibrogenic pathways. By targeting upstream metabolic derangements while concurrently limiting inflammation and fibrosis, empagliflozin may reduce the likelihood of transition to advanced stages of liver disease, including cirrhosis and HCC. Although these conclusions are derived primarily from preclinical studies, they provide a strong biological rationale for future long-term clinical trials designed to further confirm the emerging and potential therapeutic uses of empagliflozin (Figure 34).

### Study limitations

Despite the diverse promising findings demonstrated in this meta-analysis, several limitations were encountered in our meta-analysis. First, the high heterogeneity between the studies included, which is a common finding in preclinical studies due to high variation in animal species, modelling techniques, differences in experimental designs, and drug dosage. Although subgroup analysis was done according to species, modelling method, and dosage, a substantial degree of heterogeneity was still present in most studies, which may be attributed to unidentifiable factors. So, we recommend that the results of the variables with high heterogeneity be considered with caution. Second, some studies, particularly Heo et al. and Lee et al., exhibited a high standard deviation of means. To investigate the possible effects of this high SD, we conducted a sensitivity analysis excluding Heo et al. and Lee et al.; the result was that the overall direction of effect remained consistent.

Third, the lack of standardized fibrosis staging across the included studies. The majority of the in vivo models investigated MASLD as a general pathology and relied primarily on qualitative histological assessments using various tissue stains, rather than formal quantitative staging systems. We directly contacted the corresponding authors for more data if available, and we successfully obtained data from Kim et al. Fourth, defective methodological quality in many of the included studies; lack of detailed reporting of allocation concealment, blinding of experiments, and randomization for outcome assessment, raising some concerns regarding the internal validity of these studies. Fifth, the limited number of studies, the small sample size, and the relatively short duration of the modelling techniques may substantially affect their translational applicability to human beings. These limitations highlight the need for more standardized preclinical studies to strengthen their translational applicability to human MASLD. Finally, presentation of data in some studies in the form of graphs rather than numerical values, to overcome this, we sent e-mails to the related authors to obtain deficient data. Whenever we found no response, we extracted numerical data from the graphs by two independent researchers using WebPlotDigitizer 4.7. Any difference in estimations was checked and corrected by the research team leader.

## Conclusion

This systematic meta-analysis showed that empagliflozin protected the liver against hepatic accumulation of lipids, together with a significant reduction of hepatic oxidation, inflammation, and fibrosis. This finding addresses the promising role of empagliflozin in the prevention and treatment of human MASLD and its associated metabolic dysregulation.

## Future perspectives

Tissue fibrosis and subsequent extracellular matrix deposition develop over extended periods. Because most of the studies included in our meta-analysis were conducted over a limited timeframe, the full anti-fibrotic capacity of empagliflozin remains to be fully elucidated. Future research should include more homogeneous studies with longer-duration experimental models to allow for better assessment of the long-term molecular and structural alterations associated with empagliflozin treatment in MASLD. Moreover, more studies are needed to elucidate the interactive role of the spleen in the pathogenesis and development of MASLD and MASH.

## Data Availability

The original contributions presented in the study are included in the article/supplementary material, further inquiries can be directed to the corresponding author.
